# PRY-1/Axin signaling regulates lipid metabolism in *Caenorhabditis elegans*

**DOI:** 10.1371/journal.pone.0206540

**Published:** 2018-11-07

**Authors:** Ayush Ranawade, Avijit Mallick, Bhagwati P. Gupta

**Affiliations:** Department of Biology, McMaster University, Hamilton, Ontario, Canada; University of Illinois, UNITED STATES

## Abstract

The nematode *Caenorhabditis elegans* constitutes a leading animal model to study how signaling pathway components function in conserved biological processes. Here, we describe the role of an Axin family member, PRY-1, in lipid metabolism. Axins are scaffolding proteins that play crucial roles in signal transduction pathways by physically interacting with multiple factors and coordinating the assembly of protein complexes. Genome-wide transcriptome profiling of a *pry-1* mutant revealed differentially regulated genes that are associated with lipid metabolism such as vitellogenins (yolk lipoproteins), fatty acid desaturases, lipases, and fatty acid transporters. Consistent with these categorizations, we found that *pry-1* is crucial for the maintenance of lipid levels. Knockdowns of *vit* genes in a *pry-1* mutant background restored lipid levels, suggesting that vitellogenins contribute to PRY-1 function in lipid metabolic processes. Additionally, lowered expression of desaturases and lipidomic analysis provided evidence that fatty acid synthesis is reduced in *pry-1* mutants. Accordingly, an exogenous supply of oleic acid restored depleted lipids in somatic tissues of worms. Overall, our findings demonstrate that PRY-1/Axin signaling is essential for lipid metabolism and involves the regulation of yolk proteins.

## Introduction

Axin was identified initially as a negative regulator of the WNT-signaling pathway [1]. Subsequently, Axin family members were shown to also be essential in numerous developmental events including embryogenesis, axis formation, cell differentiation, and tissue homeostasis [2–5]. As a scaffolding protein Axin plays a key role in the regulation of canonical WNT pathway function. It contains multiple domains that facilitate homodimerization and interactions with the destruction-complex proteins Dishevelled, APC, and GSK-3β [4, 6]. In turn, the destruction complex initiates the phosphorylation and consequent proteolysis of the transcriptional regulator β-Catenin, which promotes expression of WNT target genes [4, 6]. Constitutive activation of β-Catenin, consequent to the loss of destruction-complex function, is often associated with cancers and various other disorders affecting the lungs, heart, muscles, and bones [4]. Thus, Axin function is crucial toward ensuring precise regulation of β-Catenin-mediated WNT signaling. In addition to WNT pathway components, Axin homologs also interact with various other factors such as Smad3, MEKK4, and LKB1 to affect diverse processes, for example cell proliferation, metabolic homeostasis, and tissue aging [7–10]. However, the mechanism of Axin function in these processes is not fully understood.

In *Caenorhabditis elegans*, PRY-1 is a member of the Axin family that is involved in multiple events such as cell fate specification, neuronal differentiation, and tissue formation [11–13]. Molecular genetic experiments have shown that PRY-1 interacts with APR-1/APC and GSK-3/GSK-3β to regulate BAR-1/β-Catenin-mediated gene transcription [14]. The phenotypes of *pry-1* mutants are consistent with overactivation of WNT signaling, such as Q cell migration and vulval induction [13, 14]. Our group has been investigating *pry-1* role in developmental as well as post-developmental processes. To identify genes that interact with *pry-1*, we performed a genome-wide transcriptome profiling experiment. Among the genes that were differentially expressed many were found to be associated with lipid metabolism. Consistent with this, *pry-1* mutant animals showed reduced lipid level. We examined a subset of differentially regulated genes, specifically yolk lipoproteins vitellogenins (VITs), which are important for lipid distribution [15], in mediating *pry-1* function. Knockdowns of *vit* genes by RNA interference (RNAi) rescued lipid defects in *pry-1* mutants. We also found that the expression of three conserved stearoyl-CoA-desaturases, *fat-5*, *fat-6*, and *fat-7*, which are involved in the synthesis of monounsaturated fatty acids such as oleic acid (OA) [16–18], was reduced in *pry-1* mutants. In support of this, supplementing the bacterial diet of mutant animals with OA partially rescued the lipid phenotype. These results provide evidence for the important role of PRY-1 in lipid metabolism through the regulation of vitellogenesis.

## Materials and methods

### Strains

Worms were grown on standard Nematode Growth Medium (NGM) agar plates using procedure described previously [19]. Cultures were maintained at 20 °C unless mentioned otherwise. The *mu38* allele of *pry-1* was obtained from CGC. All strains were outcrossed at least three times before doing the experiments.

The genotypes of the strains used in this study are: N2 (wild-type), DY220 *pry-1(mu38) I*, DY656 *pry-1(mu38) I; ldrIs2[mdt-28p*::*mdt-28*::*mCherry]*, EW15 *bar-1(ga80) X*, LIU2 *ldrIs2[mdt-28p*::*mdt-28*::*mCherry]*, PS4943 *huIs[dpy-20;hsp16-2*::*dNT-bar-1]; syIs148[pT00*.*49 + unc-119(+)]*, RB1982 *vit-1(ok2616) X*, RB2365 *vit-2(ok3211) X*, RB2202 *vit-4(ok2982) X*, RB2382 *vit-5(ok3239) X*, STE68 *nhr-49(nr2041) I*, STE70 *nhr-80(tm1011) III*.

### Heat shock treatments

For lipid quantification, *hsp-16-2*::*dNT-bar-1* (termed as *hs*::*dNT-bar-1*) animals were age synchronized and grown at 20 °C till young adult after which they were heat treated at 38 °C for 30 min or 30 °C for 12 hours. Subsequently, the animals were incubated at 20 °C for one hour before carrying out lipid staining. For qPCR analysis, *hs*::*dNT-bar-1* eggs were incubated for 16 hours. The hatched L1 animals were heat treated at 38 °C for 30 min. They were then allowed to recover for 90 min at 20 °C prior to extraction of RNA.

### Molecular Biology

For qRT-PCR experiments mRNA was extracted from bleach synchronized worms by Tri-reagent (Catalog Number T9424, Sigma-Aldrich Canada) according to the manufacturer’s instructions. cDNA was synthesized from total RNA using oligo (dT) primers and other reagents in the ProtoScript First Strand cDNA Synthesis Kit (Catalog Number E6300S, NEB, Canada). Quantitative real-time PCR (qRT-PCR) analysis was performed on a CFX 96 BioRad cycler in triplicate with SensiFAST SYBR Green Kit (Catalog Number BIO-98005, USA), according to manufacturer’s instructions. *pmp-3* was used as a reference gene in all assays. CFX manager was used for the Ct and *p*-value calculations. The primers used in this study are listed in S1 Table.

### RNAi

For RNAi experiments, *Escherichia coli* HT115 expressing target specific dsRNA were grown on plates containing β-lactose [20]. Worms were age synchronized by bleach treatment and seeded onto plates. After becoming young adults, worms were transferred to fresh plates every other day and the numbers of dead worms were recorded. For adult specific RNAi, synchronized worms were cultivated on NGM/OP50 plates until the young adult stage and then transferred to the RNAi plates.

### Lipid analysis by Oil Red O staining and fluorescence measurements

Oil Red O (Sigma-Aldrich, Canada, Catalog number O0625-25G**)** staining was performed as previously reported [21]. Briefly, worms were collected from NGM plates, washed with 1x phosphate-buffered saline (PBS) buffer (pH 7.4), and re-suspended in 60 μl of 1x PBS, 120 μl of 2x MRWB buffer (160 mM KCl, 40 mM NaCl, 14 mM Na_2_-EGTA, 1 mM spermidine-HCl, 0.4 mM spermine, 30 mM Na-PIPES [Na-piperazine-*N*, *N*′-bis (2-ethanesulfonic acid); pH 7.4], 0.2% β-mercaptoethanol), and 60 μl of 4% paraformaldehyde. The worms were then freeze-thawed three times and washed twice with 1x PBS. They were then incubated at room temperature in 60% isopropyl alcohol for 10 minutes for dehydration and stained with freshly prepared Oil Red O solution for at least 48 hours on a shaker. For direct and consistent comparison, all Oil Red O images from the same experiment were acquired under identical settings and exposure times. Animals were mounted and imaged using Q-imaging software and a Micropublisher 3.3 RTV color camera outfitted with DIC optics on a Nikon 80i microscope. NIH ImageJ software (https://imagej.nih.gov/ij/) was used to quantify Oil Red O intensities as described previously [21]. A total of 15 to 30 worms were randomly selected from each category in at least two separate batches.

For lipid quantification using fluorescent mCherry marker, animals were paralyzed in 10 mM Sodium Azide and mounted on glass slide carrying 2% agar pads. Images were acquired using NIS Element software (Nikon, USA) and a Hamamatsu Camera attached to a Nikon Eclipse 80i upright Nomarski microscope. At least two batches of animals, each containing 20 or more, were examined. All images were acquired under fixed software settings. Quantification of pixel densities was performed using Image J.

### Brood assay

Worms were bleach synchronized and allowed to grow to L4 stage for determining the progression of egg-laying and the brood size. Individual worms were picked onto a separate NGM plate with *E*. *coli* OP50 bacteria and allowed to grow for several days. Worms were transferred routinely to freshly seeded NGM plates and progeny were counted every 24 hours. Data from escaping or dying mothers were omitted from the analyses [22].

### Oleic acid supplementation assay

To make Oleic acid (OA) supplemented NGM agar plates, a 0.1 M water-based stock solution of OA sodium salt (NuCheck Prep, USA, Catalog number U-46-A) was prepared and stored at –20 °C in the dark. The OA solution was added continuously to the NGM and promptly poured into the plates. The plates were covered with aluminum foil and kept at room temperature overnight to dry. The *E*. *coli* OP50 strain was seeded onto each plate and allowed to further dry for one to two days in the dark. Oil Red O staining was performed as described above [23].

### Lipase assay

Lipase activity was estimated using commercially available QuantiChrom Lipase Assay Kit (BioAssay Systems, USA, Catalog number DLPS-100) and processed according to the manufacturer’s instructions. 1 unit of Lipase catalyzes the cleavage of 1 μmol substrate per minute. Three independent samples of one-day-adult worms were prepared by homogenizing in a solution (20% glycerol, 0.1 M KCl, 20 mM HEPES pH 7.6). Measurements were done as described earlier [24].

### L1 survival assay

Worms were bleach synchronized and kept in a 1.5 ml centrifuge tube. The progeny were seeded onto NGM plates approximately 24 hours later and transferred regularly for 12 days. Worms were grown to young adult stage before counting the survivors. The data was statistically compared using an analysis of covariance (ANCOVA) model.

### RNA-Seq and data analysis

*pry-1* targets were examined in synchronized L1 stage animals. At this stage WNT ligands, receptors, and targets are highly expressed as revealed by microarray studies from SPELL database [25, 26] (S1A–S1D Fig). Also, our qRT-PCR experiments showed significant upregulation of three of the WNT targets, *lin-39*, *egl-5* and *mab-5*, in *pry-1* mutants at L1 stage (S1D Fig). The *pry-1* transcriptome profile can be found in the GEO archive with accession number GSE94412. For RNA-Seq experiments synchronized L1 stage animals were obtained by two successive bleach treatments and RNA was isolated using Trizol-reagent (Sigma, USA, Catalog Number T9424) [27]. The quality of total purified RNA was confirmed using bioanalyzer (Agilent 2100 and Nanodrop 1000). cDNA libraries were constructed from 100–200ng mRNA using an Illumina-specific commercial kit (TruSeq RNA Sample Preparation Kit v2, Set A, Catalog number RS-122-2001). RNA sequencing was carried out using Illumina Hi-Seq 2000 system at the McGill University Genome Quebec sequencing facility. For each of the N2 and *pry-1(mu38)* strains two biological replicates were used. For each cDNA library, 100 bp paired-end reads were obtained. In total, 30 to 38 million reads were obtained for each sample analyzed for differential gene expression.

The adapters were trimmed using cutadapt/trimgalore, reads with QC values (Phred score) lower than 30 bases were discarded after trimming [28]. Later, processed sequencing reads were mapped to the reference genome (ce6) (UCSC 2013) using the software package Bowtie 1.0.0 [29]. 92–95% of total sequenced fragments could be mapped to the genome (S2 Table). Transcript-level abundance estimation was performed using eXpress 1.5 software package [30]. Among all genes analyzed, we were able to map 18,867 to known transcripts by at least one sequencing fragment in *C*. *elegans*. To avoid biases between samples, the gene counts were quantile normalized [28, 31]. Using a negative binomial distribution model of DESeq package in R, differentially-expressed genes were called at a false discovery rate (FDR) of 0.05% [32].

GO analysis was carried out with default setting using GoAmigo (http://amigo.geneontology.org). A GO-term containing at least three genes with a *p*-value adjusted for multiple comparisons and < 0.05 (Benjamini-Hochberg method) was counted significant [33]. Tissue enrichment analysis was performed using Wormbase online TEA tool that employs a tissue ontology previously developed by WormBase [34].

### Gas chromatography mass spectrophotometry (GC-MS)

We modified the fatty Acid analysis protocol from a previously published method [16, 35]. Eppendorf tubes, glass vials or any containers used for the extraction process were sonicated in dichloromethane (Caledon Laboratories Ltd., Canada, Catalog number 3601-2-40) for 30 minutes to eliminate lipid contamination. To determine FA composition, we collected few thousand adult worms from three 6-cm plates and removed residual bacteria by washing the animals with sterile water. Washed worms were placed into a screw-capped centrifuge tube and spun at 2,500 RPM for 45 seconds. The residual water was removed using a Pasteur pipette and worms were transferred to glass vials (Agilent, part number 5182–0714) and accurately weighed to 50–100 mg. Fatty acids were then extracted from tissues and transmethylated by adding 1 ml of 2.5% H_2_SO_4_ (Caledon Laboratories Ltd., Canada, Catalog number 8825-1-05) diluted in methanol (Caledon Laboratories Ltd., Canada, Catalog number 6701-7-40). We spiked the samples with 10 μl of a recovery standard (stearic acid 120 ng/μl, Sigma-Aldrich Canada, Catalog number S4751-1G) and incubated at 80 °C for an hour in a water bath. To this, a mixture of 0.2 ml of hexane (Caledon Laboratories Ltd., Canada, Catalog number 3201-7-40) and 1.5 ml of water was added and slowly spun to extract fatty acid methyl esters into the hexane layer. Agilent 6890 series gas chromatographer equipped with a 30 × 0.25 mm SP-2380 fused silica capillary column (Supelco USA), helium as the carrier gas at 1.4 ml/minute, and a flame ionization detector was used for fatty acid analysis. Automatic injections of 1 μl samples in the organic phase were made, without splitting, into the GC inlet set to 250 °C. The thermal program began at 50 °C for 2 minutes, then increased linearly to 300 °C at a ramping rate of 8 °C/minute and held this temperature for 15 minutes. A constant flow rate of 1 ml/minute helium carrier gas under electronic pressure control was maintained for the fatty acid composition determination by TIC method using standard software. For quantitation of fatty acids, the peaks across all GC-MS runs were aligned using both chromatographic information (retention times) and mass-spectral data (m/z components) to establish the chemical identity of peaks being compared. We calculated the relative fatty acid amounts by dividing each peak area by the sum of areas for all fatty acid peaks appearing on the same chromatogram. For each fatty acid, the quantities determined by GC-MS were successively normalized in two ways: 1) to an internal standard naphthalene-d8, 10 ng/μl (1 ng/μl in injection sample) added to each sample prior to sonication and lipid extraction), and 2) to the weight of the samples.

### Statistical analysis

The statistics were performed using Microsoft Office Excel 2016. If not specifically mentioned, *p* values for the fertility, motility, fat content, L1 survival, and enzyme activities were calculated using the Student’s *t* test after testing for equal distribution of the data and equal variances within the data set. Experiments were performed in triplicates except where stated otherwise. Differences were considered statistically significant at *p* < 0.05, thereby indicating a probability of error lower than 5%. Hypergeometric probability tests and statistical significance of the overlap between two gene sets were done using an online program (http://nemates.org/MA/progs/overlap_stats.html).

## Results

### Identification of PRY-1 targets

**Fig 1:**
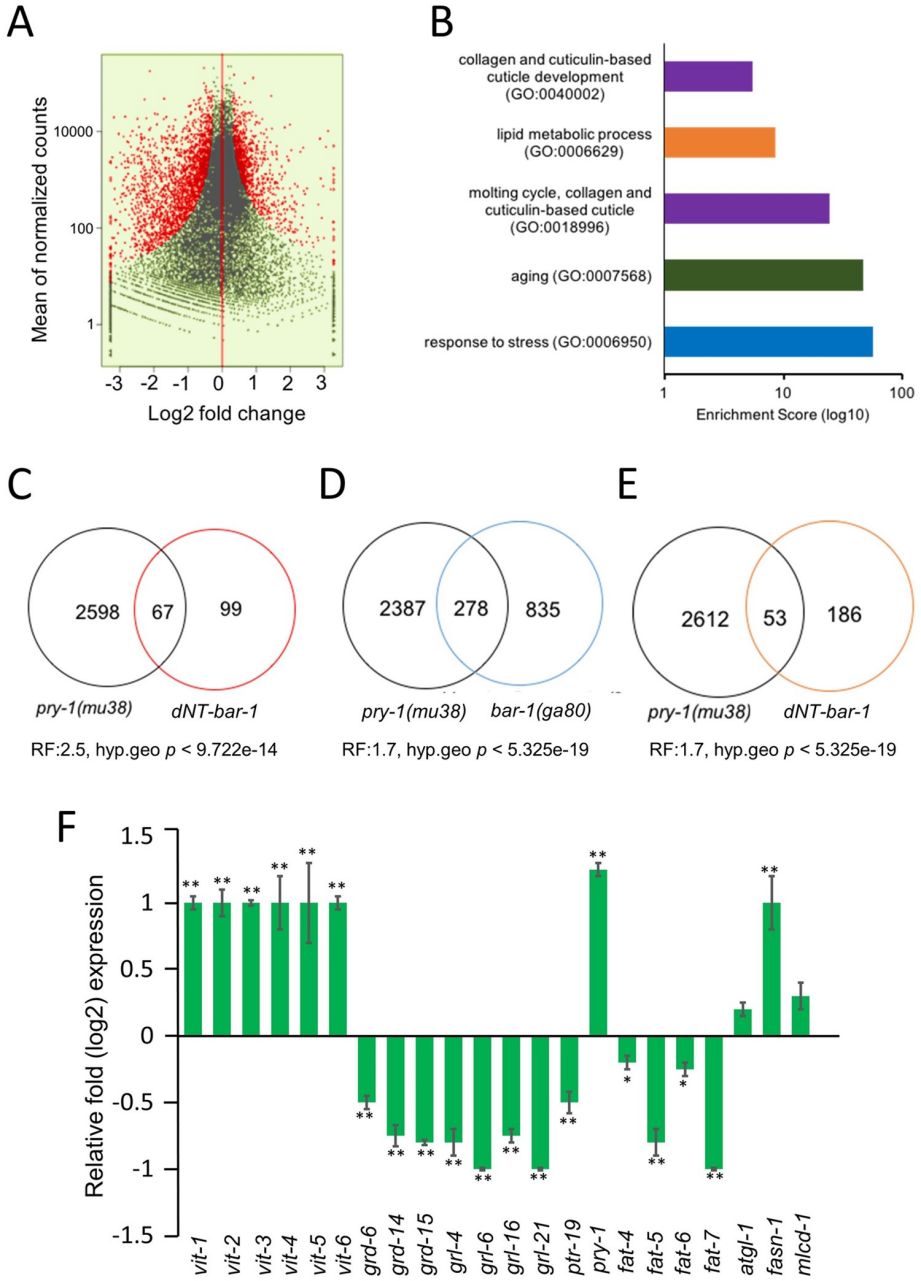
*pry-1(mu38)* transcriptome analysis. (A) Scatter plots of differentially expressed genes in *pry-1(mu38)*. Red dots mark significantly altered transcripts with a FDR *p*-adj of < 0.05, whereas black dots mark transcripts that are not significantly altered (FDR *p*-values of > 0.05). (B) Selected GO categories enriched in *pry-1(mu38)* targets are identified by GO Amigo, (*p*-adj < 0.05). (see S4 Table for a detailed list). (C-E) Venn diagrams showing the overlap between *pry-1(mu38)*, *hs*::*dNT-bar-1*, and *bar-1(ga80)* transcriptional targets. P values are shown in each case. (C) Overlap between *pry-1(mu38)* and *hs*::*dNT-bar-1*whole animal transcriptome data [36]. (D) Overlap between *pry-1(mu38)* and *bar-1(ga80)* whole animal transcriptome data [37]. (E) Overlap between *pry-1(mu38)* and *hs*::*dNT-bar-1* vulva and seam cell-specific transcriptome data [38]. (F) qPCR analysis of selected genes in the *pry-1(mu38)* mutant. Data represents the mean of at least two replicates and error bar represents the SEM. *p < 0.05, **p < 0.01.

To gain insights into the mechanism of *pry-1*-mediated signaling, a genome-wide transcriptome analysis was carried out to identify the potential downstream targets. The RNA-Seq was performed in *pry-1* mutant animals carrying a nonsense allele, *mu38*, that causes constitutive activation of WNT signaling [14]. We identified a total of 2,665 genes (767 upregulated and 1898 downregulated, False Discovery Rate (FDR) *p*-adj 0.05) that were differentially expressed in *pry-1(mu38)* animals during the L1 larval stage (Fig 1A, S3 Table, also see Methods). Of these, the transcription of 1,331 genes was altered twofold or more (FDR, *p*-adj 0.05) (248 upregulated and 1083 downregulated) (S3 Table). The average and median fold changes in the expression were 2.2 and 2.0, respectively. Fig 1A shows a scatter plot of all expressed genes. A subset of these genes was also tested by quantitative polymerase chain reaction (qPCR), which revealed an 85% validation rate (Fig 1F).

We next carried out gene ontology (GO) analysis (www.geneontology.org) to investigate the processes affected in *pry-1(mu38)* animals. Genes with altered expression were found to be enriched in GO terms associated with “determination of adult lifespan”, “aging”, “response to unfolded protein”, “oxidation-reduction process”, “metabolism”, “stress response and cell signaling”, “steroid hormone mediated signaling”, “lipid metabolic processes”, and “cellular response to lipids” (Fig 1B; a complete list is provided in S4 Table). This suggests that *pry-1* plays a role in stress response, lipid metabolism, and lifespan regulation. We also observed enrichment in neuron-related GO terms such as “axon”, “synapse”, “synaptic transmission”, and “neuron development”, which was expected from the requirements of *pry-1* in neuronal development [14]. Other categories included “molting cycle”, “regulation of transcription”, “DNA-template”, and the “reproductive process”. In addition to these known categories, the dataset included numerous non-annotated genes (S3 Table) whose functions remain uncharacterized.

Analysis of genes and gene families revealed that several components of Hedgehog (HH) signaling are downregulated in *pry-1(mu38)* animals including warthog genes *wrt-1* and *wrt-9*; groundhog-like genes *grl-1*, *grl-4*, *grl-5*, *grl-6*, *grl-7*, *grl-13*, *grl-16*, and *grl-21*; hedgehog-like genes *grd-3*, *grd-5*, *grd-12*, *grd-14*, and *grd-15*; and patched-related genes *daf-6*, *ptr-1*, *ptr-13*, *ptr-16*, *ptr-19*, *ptr-2*, *ptr-20*, *ptr-21*, *ptr-22*, *ptr-23*, and *ptr-8*. This suggests that *hh*-signaling is affected by *pry-1*. Some of these genes were also recovered earlier in *bar-1* transcriptome studies [36–38], discussed further below. The *ptc* and *ptr* genes promote molting and the trafficking of proteins, sterols, and lipids [39, 40]. In support of such function, we found molting and other cuticle-related defects in *pry-1* mutants (e.g., rollers, defective alae, and weaker cuticle) (Mallick *et al*., manuscript in preparation). These data are consistent with the role of WNT signaling in cuticle development [36].

Alterations in the expression of some of the WNT pathway components were noted as well. For example, *pry-1* was up 1.3 fold (on log2 scale, S3 Table, Fig 1F). Although such upregulation of *pry-1* was not reported previously in *pry-1* mutants, prior studies have shown that Axin constitutes a target of WNT signaling and its expression is increased in overactivated WNT backgrounds [6, 36, 38, 41]. Thus, positive regulation of Axin by the canonical WNT signaling represents a conserved mechanism in eukaryotes. Other WNT pathway components that were differentially expressed in *pry-1(mu38)* included *mom-2/wnt* (1.5-fold increase), *cfz-2/fz* (1.7-fold decrease), *lin-17/fz* (1.6-fold increase), and *pop-1/tcf* (1.7-fold increase) (S3 Table).

A comparison of *pry-1* RNA-Seq dataset with three previously reported WNT transcriptome microarray studies involving *bar-1(ga80)* [37], which is a genetic null, and constitutively active *bar-1* (*hs*::*dNT-bar-1*, heat-shock promoter-driven *bar-1* carrying a small deletion in the *N*-terminus) [13, 36, 38] revealed shared genes and families. Of the two *hs*::*dNT-bar-1* studies, one was specific to the vulva and seam cells [38] and the other involved whole animal analysis [36]. A total of 12% [38], 18% [37], and 30% [36] *bar-1* set of genes overlapped with our *pry-1* set (Fig 1C–1E, S5 Table). These include several *hh* family members. Altogether, from the three *bar-1* studies mentioned above, 10 *hh* genes, namely *grl-5*, *grl-10*, *grl-14*, *grl-15*, *hog-1*, *grd-1*, *grd-2*, *grd-12*, *wrt-4*, and *wrt-6* were reported, of which three (*grl-5*, *grl-14*, and *grd-12*) are present in our *pry-1* list. The other shared genes include those involved in cuticle synthesis (*col* and *cutl* families), defense response, embryo development, oxidation-reduction processes, and proteolysis. We also analyzed shared genes based on their expression trend, i.e., up or down, using GO terms. The analysis revealed over-representation of categories, namely immunity, stress response, and lipid catabolic processes (S6 Table), suggesting that these genes might act downstream of both PRY-1 and BAR-1.

### *pry-1* mutants exhibit altered lipid metabolism

Throughout the lifespan of an animal, lipids are persistently mobilized to supply energy demands for growth, cellular maintenance, tissue repair, and reproduction [42, 43]. Changes in lipid levels also affect an organism’s ability to survive in stressful conditions [44, 45]. Notably, many genes that are involved in the synthesis, breakdown, and transport of lipids are differentially expressed in *pry-1* mutants (S2 Fig). These include vitellogenins (yolk protein/apolipoprotein-like): *vit-1*–*6*; fatty acid transporters: *lbp-1*, *-2*, *-4*, *-5*, *-7*, and *-8*; lipases: *lips-3*, *-4*, *-7*, *-10*, and *-17*; desaturases: *fat-4-6*; elongases: *elo-3* and *-6*; and fatty acid oxidation: *acdh-1*, -6, *-11*, *-23*, *acs-2*, *-11*, and *-17*, *cpt-1*, *-4*, and *ech-9* (S2 Table). The expression of *vit* genes and desaturase was measured by qPCR and all but *fat-4* were successfully validated (Fig 1F) as levels of the latter were decreased by 20%, unlike the 1.5-fold increase observed by RNA-Seq. We also tested another desaturase, *fat-7*, which functions redundantly with *fat-6* [46] but was not present in our dataset. The *fat-7* mRNA level was significantly reduced (Fig 1F); thus, all four *fat* desaturase genes are downregulated in *pry-1(mu38)* animals.

**Fig 2:**
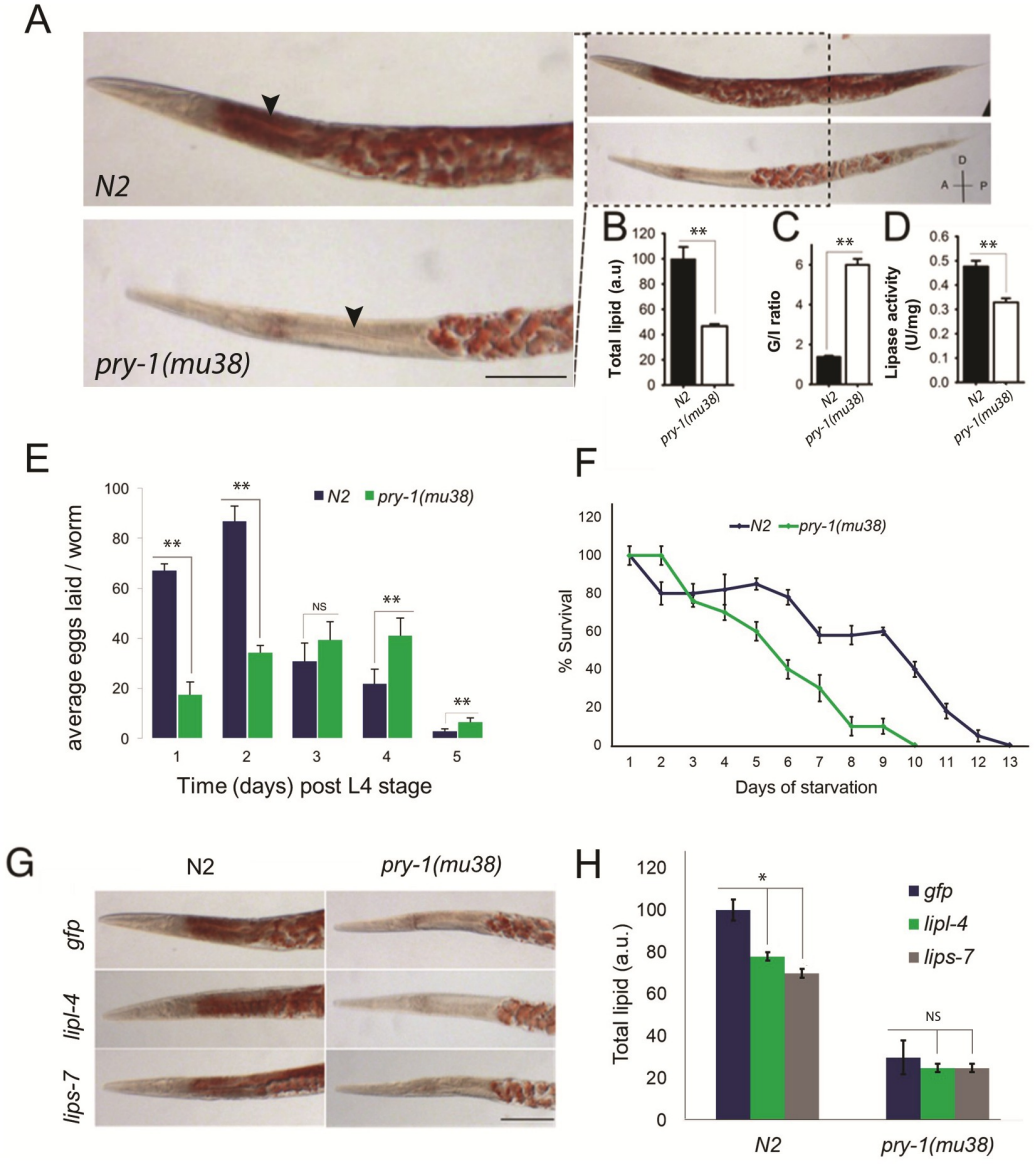
Lipid levels and distribution are altered in *pry-1(mu38)* mutants. Arrowheads indicate intestine and dotted areas gonad. (A) Representative DIC images of N2 and *pry-1(mu38)*, stained with Oil Red O. D: dorsal; V: ventral; A: anterior; and P: posterior. (B, C) Quantification of Oil Red O staining. Data represents the mean of at least two replicates and error bar in this and subsequent graphs represents the SEM. (C) Ratio of gonadal to intestinal (G/I) lipid. (D) The lipase activity is decreased in *pry-1(mu38)*. The activity per mg of protein has been plotted. (E) The average number of eggs laid by wild-type and *pry-1(mu38)* animals on different days over the duration of their reproductive period. (F) *pry-1(mu38)* displayed significant reduction in L1 survival following starvation. Percent survival of L1 larvae in the absence of food has been plotted. Graph represents the average of three independent experiments. (G) Representative images of N2 and *pry-1(mu38)* RNAi-treated animals stained with Oil Red O**.** (H) Histograms show Oil Red O intensities. Scale bar, 50 μm. *p < 0.01, **p < 0.001.

**Fig 3:**
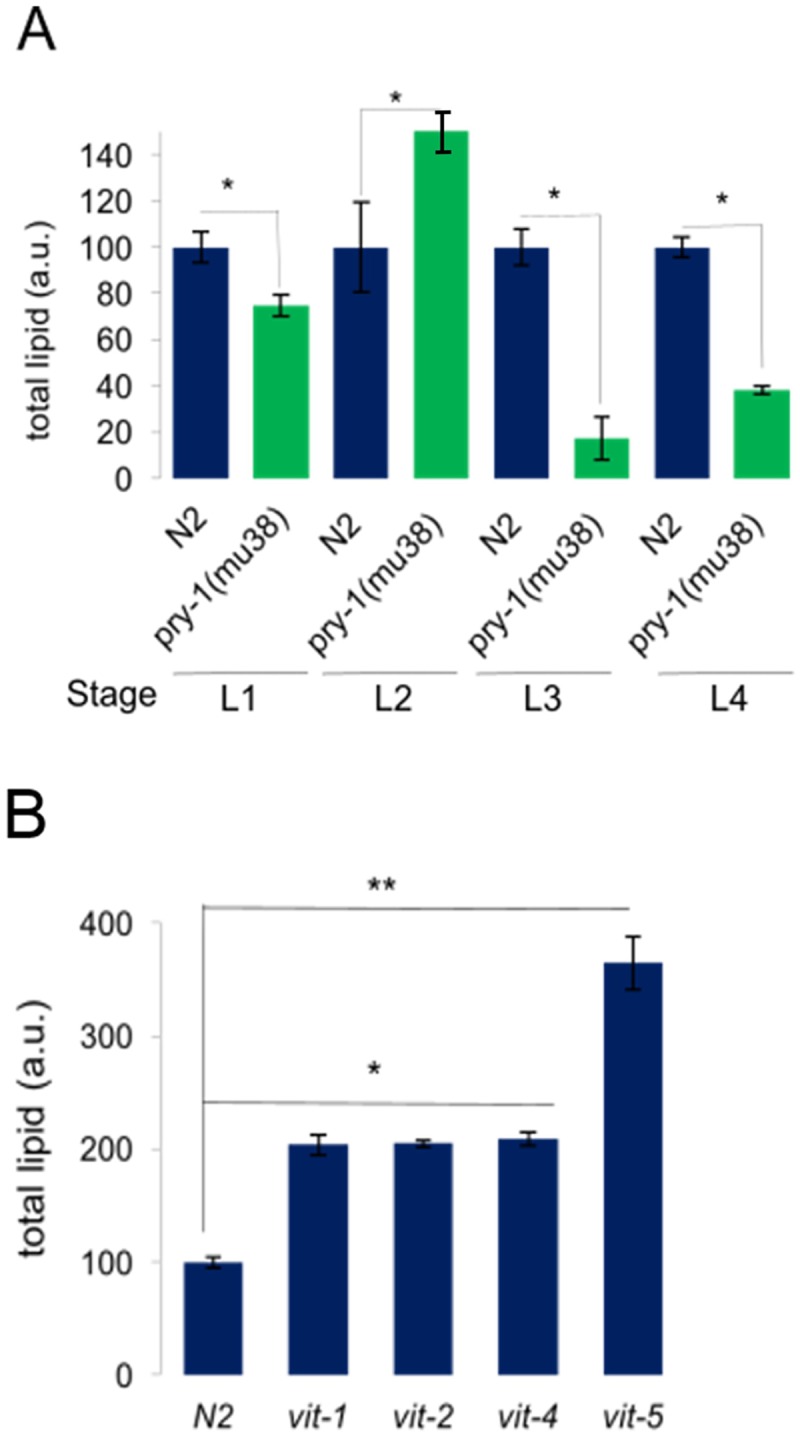
Lipid levels in *pry-1(mu38)* and *vit* mutants. (A) Quantification of Oil Red O staining intensity in *pry-1(mu38)* and wild-type animals at different developmental stages. Lipids are lower in *pry-1* mutants at all stages except L2. (B) Quantification of Oil Red O staining in N2 and *vit* mutants during the young adult stage. Lipid levels are higher in *vit-1(ok2616)*, *vit-2(ok3211)*, *vit-4(ok2982)*, *and vit-5(ok3239)* animals. Data represents the mean of at least two replicates and error bar represents the SEM. *p < 0.01, **p < 0.001.

Enrichment of several lipid metabolism genes in the *pry-1* transcriptome led us to examine lipid accumulation in worms. Staining with Oil Red O revealed that the lipid content was less than half in *pry-1(mu38)* one-day-old young adults compared with controls (Fig 2A and 2B). Examination of total fat at each larval stage revealed that *pry-1* mutants had lower somatic lipid stores (25–80%) at all stages except for L2 (Fig 3A). In addition, the lipid distribution was altered such that the staining was mostly restricted to gonadal tissue (Fig 2C). These results suggested that *pry-1* plays a role in lipid metabolism. Consistent with this model, we found that *pry-1(mu38)* animals laid fewer fertilized eggs and had poor survival upon starvation-induced L1 diapause (Fig 2E and 2F).

One explanation for the reduced lipid phenotype may be that lipids are being rapidly utilized. However, this is unlikely because several lipases (*lips* family members) were downregulated. We also measured lipase activity in one-day-old adults from whole worm lysates. As expected, the total activity was 34% lower in the mutant compared with the N2 control (Fig 2D). Next, we examined lipids in *pry-1(mu38)* animals following knockdown of *lipl-4* or *lips-7*, which comprise lipase genes that regulate the gonad-dependent somatic lipid levels [42, 43, 47] but observed no change in the pattern of lipid distribution (Fig 2G and 2H). We concluded that the lower somatic lipids in animals lacking *pry-1* function were not due to increased utilization, raising the possibility of the involvement of other metabolic processes.

### Vitellogenins contribute to lipid metabolism defects in *pry-1* mutants

**Fig 4:**
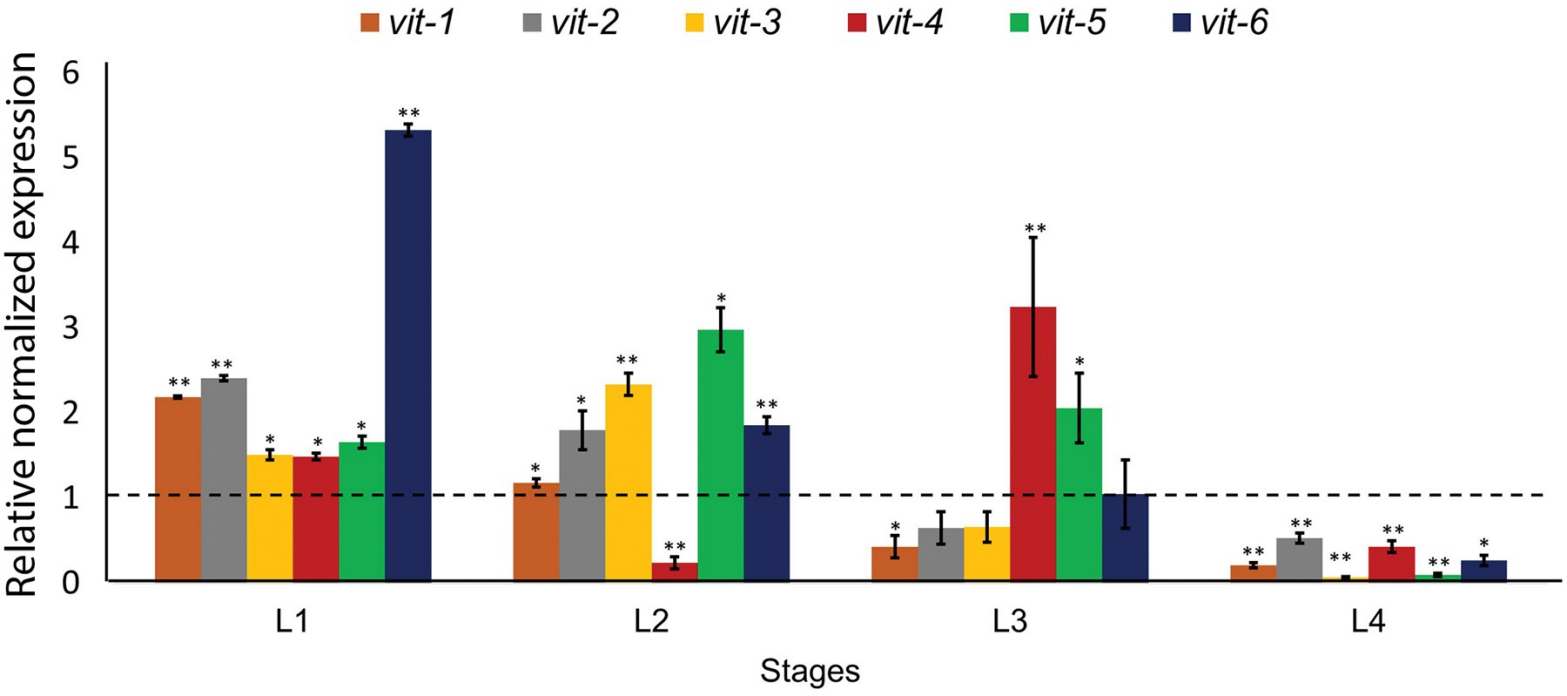
Expression of *vit* genes in *pry-1(mu38)*. (A) qRT-PCR of *vit* genes at larval stages in *pry-1(mu38)* mutants. Relative normalized expression has been plotted. Data represents the mean of at least two replicates and error bar represents the SEM. *p<0.05 and **p<0.01, compared to wild-type which is normalized to 1.

To understand the molecular basis of low lipid levels in *pry-1(mu38)* worms we focused on the vitellogenin family of genes, whose expression is repressed by *pry-1*. VITs comprise the major yolk proteins in *C*. *elegans*, which are synthesized in the intestine and mediate lipid transport from the intestine to the gonad during the reproductive period [15]. Examination of *vit* levels in *pry-1(mu38)* animals revealed abnormal expression at all developmental stages. Thus, beginning with the L1 stage where all six *vit* genes were upregulated, the number of overexpressed genes was five in L2, two in L3, and zero in L4 stage (Fig 4).

**Fig 5:**
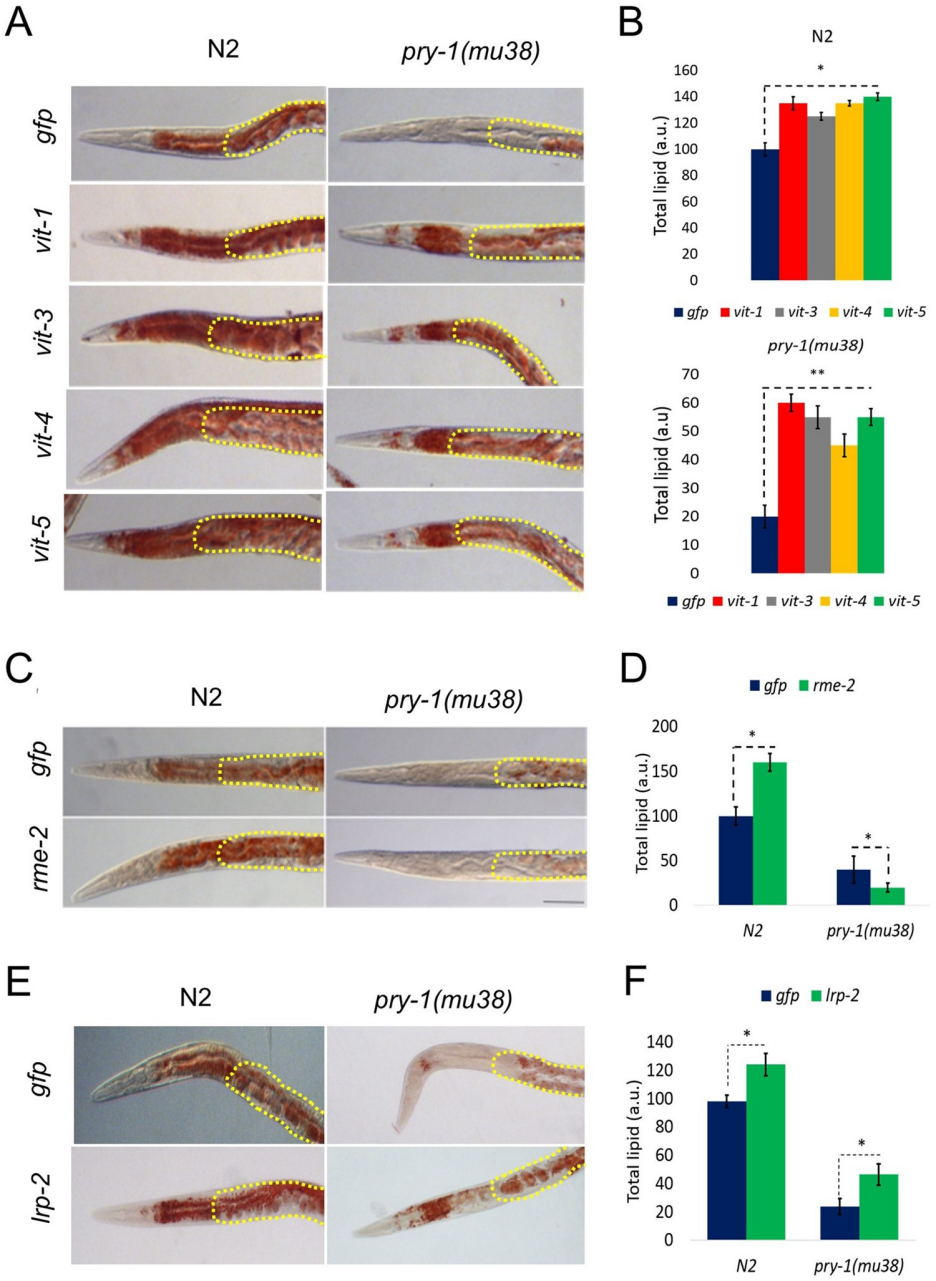
Vitellogenin mediated lipid metabolism in *pry-1* mutants. (A) DIC micrographs of representative N2 and *pry-1(mu38)* adults stained with Oil Red O following *vit* RNAi. Areas marked with dotted lines show germline and eggs. (B) The corresponding Oil Red O quantifications following each treatment. (C) Representative images of N2 and *pry-1(mu38)* following *rme-2* RNAi knockdown. (D) The histogram shows Oil Red O intensity in N2 and *pry-1(mu38)*. (E) Representative images of N2 and *pry-1(mu38)* following *lrp-2* RNAi knockdown. (F) The histogram shows Oil Red O intensity in N2 and *pry-1(mu38)*. Data represents the mean of at least two replicates and error bar represents the SEM. At least 50 animals were quantified in each batch. *p < 0.01, **p < 0.001.

We next examined lipid contents in worms using mutants and RNAi knockdowns of specific *vit* genes. The results revealed altered lipid distributions in all cases such that lipids accumulated at higher levels in somatic tissues (Figs 3B, 5A and 5B, S3A Fig). A quantification of overall lipids following *vit* knockdowns revealed a significantly higher accumulation in mutants compared to wild-type (2.5–3 fold and 1.3–1.4 fold, respectively, using *gfp* RNAi knockdown as a control in each case) (Fig 5A and 5B). A similar observation was made using the *mdt-28p*::*mCherry* reporter (S3B–S3D Fig) that marks lipid droplets [48]. Lipid accumulation following *vit* knockdown has also been reported in wild-type animals [49]. Sequence comparisons indicated that *vit-1* RNAi may also target *vit-2* owing to significant identity in the genomic region used to perform KDs (S7 Table), which was validated by qPCR (S4 Fig). Similarly, any one of the *vit-3*, *4*, or *5* RNAi could simultaneously effect knockdown of the other two (S7 Table). Thus, multiple VITs appear to play roles in the regulation of both the level as well as the distribution of lipids, and their misregulation contributes to lipid metabolism defects in *pry-1* mutants.

### Lipoprotein receptors RME-2 and LRP-2 may not contribute to lipid defects in *pry-1* mutants

To understand how *pry-1* and *vit* genes might function to regulate lipid levels, we examined the involvement of *rme-2* in the *pry-1*-mediated pathway to regulate lipid accumulation as VITs are transported via the RME-2 receptor [15]. The knockdown of *rme-2* by RNAi led to intestinal accumulation and ectopic deposition of lipids (Fig 5C and 5D) consequent to blockage of yolk protein transport to the developing oocytes [15]. Specifically, *rme-2(RNAi)* animals showed an approximately 45% increase in total lipid content such that the gonad-to-somatic ratio was roughly 30% lower compared with that in controls. However, this phenotype was not observed in *pry-1(mu38)* owing to a reduction in lipid levels both in somatic and gonadal tissues (Fig 5C and 5D). These results allow us to suggest that VITs act independently of the RME-2 transport mechanism to regulate lipid metabolism in response to *pry-1* signaling. Moreover, as lipid levels are further reduced in *pry-1(mu38)* animals following *rme-2* knockdown, *rme-2* may have an unknown non-vitellogenin-mediated role in lipid accumulation. Other possibilities also exist for such a phenotype.

We also examined other VIT-interacting factors for their involvement in PRY-1-mediated regulation of lipids. Our transcriptome dataset contained one LDL-like receptor gene, *lrp-2*, which was overexpressed in *pry-1* mutants (S3 Table). It was shown previously that *lrp-2* positively regulates yolk protein synthesis [22]. To test whether lipid levels are affected by *lrp-2*, RNAi knockdown experiments were performed, which showed a small but significant rescue of the lipid phenotype in *pry-1(mu38)* animals (Fig 5E and 5F). However, as *lrp-2* knockdown in wild-type animals also caused an increase in lipids, it is unclear whether PRY-1-mediated signaling utilizes LRP-2 function to affect lipid levels.

### *pry-1* mutants show reduced fatty acid levels

**Fig 6:**
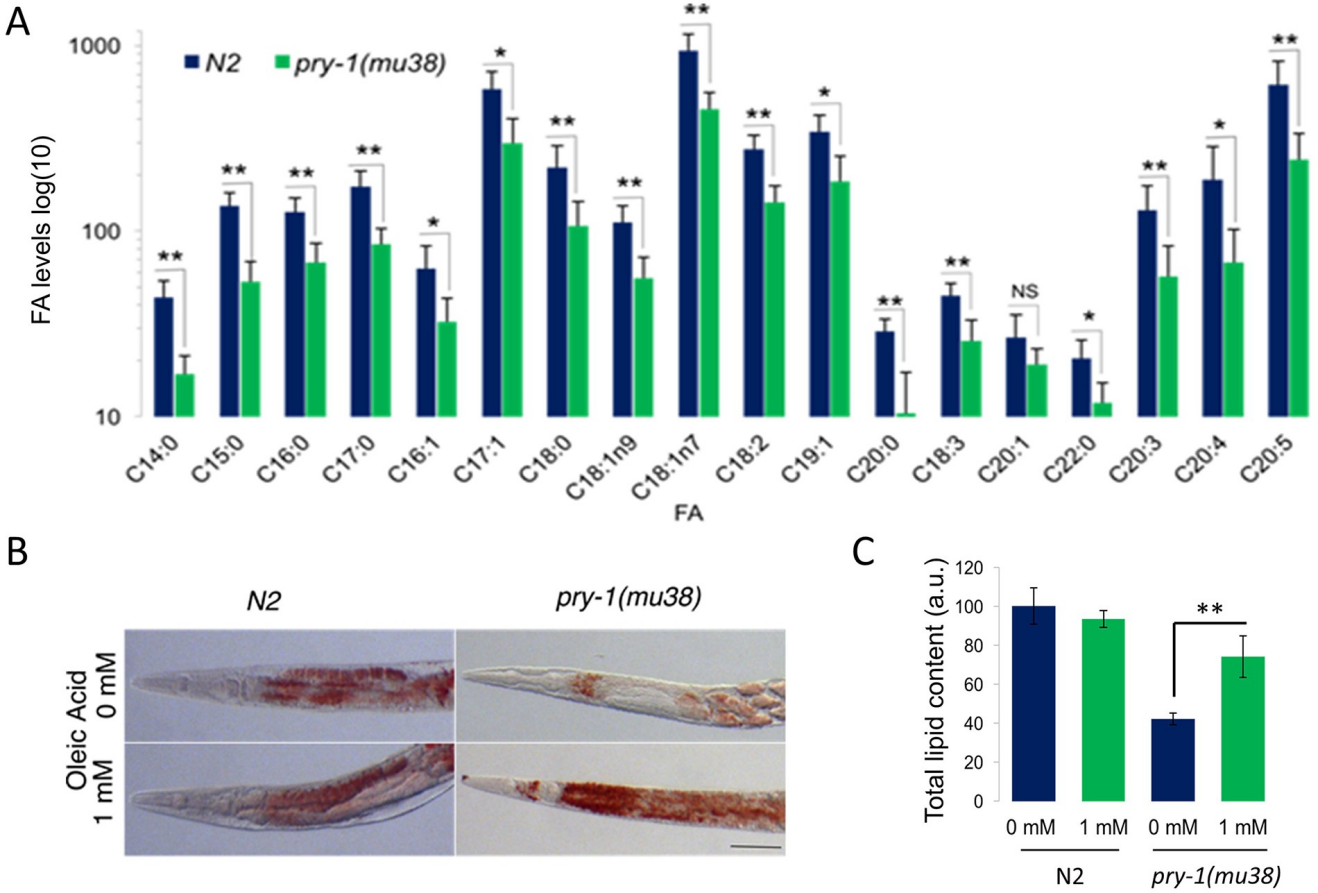
GC-MS analysis of fatty acids in *pry-1* mutants and partial rescue of lipid defects following OA treatments. (A) Total FA levels of selected fatty acid species expressed in log_10_ value as determined by GC-MS analysis. The *pry-1* mutants have low levels of FA. Error bars represent the standard deviation. Significant differences between wild type and *pry-1* mutant are marked with stars, * *p* < 0.03, ** *p* < 0.015. (B) Lipid staining of N2 and *pry-1(mu38)* adults supplemented with 1 mM OA. **C)** Quantification of total lipid, *p* < 0.001.

The *pry-1* transcriptome contains genes predicted to participate in fatty acid desaturation and elongation (5), binding/transport (6), and β-oxidation pathway (16) (S2 Fig). The Δ9-desaturase enzymes are required to produce C16:1 and C18:1 monounsaturated fatty acids (S2 Fig). Whereas *fat-5* converts palmitic acid (C16:0) to palmitoleic acid (C16:1n7), *fat-6* and *fat-7* are involved in stearic acid (C18:0) to OA (C18:1n9) conversion [17]. The downregulation of Δ9-desaturases, observed in *pry-1(mu38)* animals, may lead to reduced fatty acid synthesis. To investigate this, we quantified lipids using a gas chromatography-mass spectrometry (GC-MS) approach. The results showed that whereas the relative ratios of fatty acids in *pry-1* mutants were normal, the absolute level of each species was significantly reduced (Fig 6A, S5 Fig). The result agrees with the overall low lipid levels in *pry-1(mu38)* animals and together supports the important role of PRY-1 signaling in lipid metabolism in *C*. *elegans*.

Because the expression of *fat* genes depends on the nuclear hormone receptors *nhr-49* and *nhr-80* [18, 35], we determined levels of these NHR transcripts in *pry-1* mutant animals. Although RNA-Seq transcriptome data showed no change in either gene, qPCR revealed a subtle but significant upregulation of *nhr-80* whereas *nhr-49* was unchanged (S6 Fig). Thus, transcriptional regulation of these two NHRs is unlikely to be a mechanism affecting *pry-1*-mediated expression of Δ9-desaturase genes, although we cannot rule out the possibility that activities of one or both may be regulated post-transcriptionally in response to PRY-1 function. To further examine the involvement of *nhr* genes, two types of RNAi experiments were carried out. In one of these, *pry-1* was knocked down in *nhr-49(nr2041)* and *nhr-80(tm1011)* animals which reduced lipid levels in both mutant backgrounds (S7 Fig). In the other case, *nhr* genes were knocked down in *pry-1(mu38)* animals. While *nhr-49 RNAi* increased the lipid level likely due to *nhr-49* role in β-oxidation [35], no change was observed following *nhr-80* knockdown (S7 Fig). Overall, these results suggest that PRY-1 and the NHRs may act in parallel to regulate lipid levels.

### OA (18C:1n9) supplementation partially rescues the somatic depletion of lipids in *pry-1* mutants

OA constitutes one of the fatty acid species that showed 50% reduced levels in our GC-MS analysis (Fig 6A). OA is required for fatty acid metabolism and is synthesized by *C*. *elegans* as it cannot be obtained through the normal *E*. *coli* (OP50) diet. OA acts as a precursor for the synthesis of polyunsaturated fatty acids and triacylglycerides, which are used for fat storage [16]. The addition of exogenous OA as a fat source has been shown to rescue several fat-deficient mutants including *fat-6* and *fat-7* by restoring their fat storage, resulting in improved fertility and increased locomotion [46]. Moreover, the addition of OA in *sbp-1*, *fat-6*, and *fat-7* animals fully rescued defects in satiety quiescence [17, 18, 50]. We therefore reasoned that supplementation of OA may improve lipid levels in *pry-1(mu38)* mutants. Treatment with 1 mM OA resulted in the restoration of lipids in animals lacking *pry-1* function (up to 2-fold higher compared with the untreated control, Fig 6B and 6C). No significant changes were seen in the gonadal lipid levels, suggesting that lipid metabolism in the gonad was unaffected.

## Discussion

### PRY-1 is necessary for normal expression of genes involved in lipid metabolism

To understand the mechanism of PRY-1/Axin function, we performed transcriptome profiling on a *pry-1* mutant. The analysis revealed altered expression of many genes including those that affect hypodermis, stress-response, aging, and lipid metabolism. The hypodermal-related genes included collagens, cuticulins, and hedgehogs. Previously, expression of some of the hedgehog genes was found to be altered in *bar-1* mutants [37, 38]. Considering that cuticular defects are observed in *bar-1* [36, 38] and *pry-1* mutants (Mallick *et al*., manuscript in preparation), and that hedgehog family members play roles in cuticle shedding and the formation of alae [39], these results lead us to suggest that a genetic pathway involving *pry-1* and *bar-1* may interact with hedgehogs for normal cuticle development.

One of the key findings of our *pry-1(mu38)* transcriptome analysis was the enrichment of genes related to lipid metabolism. In particular, we found that multiple lipogenic and lipolytic genes exhibited altered expression. For example, all four fatty acid desaturases (Δ5 and Δ9 desaturases) were downregulated in *pry-1* mutants. Whereas single *fat* gene mutants affect fatty acid composition without altering overall lipid levels, double mutants have low lipid levels [17, 46], suggesting that *pry-1* positively regulates fatty acid synthesis. With regard to lipolytic genes, such as those involved in β-oxidation, changes in gene expression between peroxisomal and mitochondrial β-oxidation genes showed an opposite trend (4 of 5 upregulated and 8 of 11 downregulated, respectively). This may indicate selective utilization of long-chain over short-chain fatty acids by the *pry-1* pathway. We also observed that all four lipases (*lips* family) were downregulated including *lips-7*, which was previously shown to be involved in lifespan extension and the maintenance of lipid levels [47]. Although *lips-7* RNAi did not alter the *pry-1(mu38)* phenotype, it remains to be seen whether *pry-1* affects any of the remaining three *lips* gene(s) to modulate lipids.

In addition to lipogenic and lipolytic genes, several lipid transporters are also present in the *pry-1(mu38)* transcriptome, including two lipid-binding proteins (*lbp-5* and *lbp-8*; both downregulated), six lipoproteins (*vit-1* to *-6*; all upregulated), and a LDL-like receptor protein (*lrp-2*). Knockdown of *lbp-5* and VITs negatively affect lipid storage [51], which further emphasizes the important role of *pry-1* in the maintenance of lipids and suggests that *pry-1*-mediated signaling is involved in the utilization of lipids for energetics as well as signaling mechanisms.

### PRY-1-mediated lipid metabolism involves vitellogenins

Reduced lipids may affect tissue function and physiology in different ways; for example, owing to altered membrane structure and compartmentalization, altered signaling, reduced energy demands, and impact on autophagy. The Oil Red O staining of *pry-1(mu38)* showed a severe reduction in lipid content with a marked decline in the somatic lipid storage. It is worth mentioning that a reduced lipid phenotype was also observed in *hs*::*dNT-bar-1* animals that carry a constitutively active form of BAR-1 (S8 Fig) [13], suggesting that *pry-1* may interact with *bar-1* to maintain lipid levels.

To understand the mechanism of *pry-1* signaling in lipid regulation, we examined the possibility of increased lipid breakdown. The results showed that total lipase activity was not increased in *pry-1* mutants. Additionally, knockdowns of *lipl-4* (lysosomal lipase) and *lips-7* (cytosolic lipase), both of which negatively regulate lipid levels [42, 43], in *pry-1(mu38)* had no observable effect. Thus, selective and rapid lipid catabolism does not appear to be a factor in lipid depletion in the absence of *pry-1* function.

We then investigated the role of VIT proteins, which are the distant homologs of human apolipoprotein B [52], in maintaining lipid levels. As major yolk proteins, VITs are involved in somatic mobilization of lipids to the developing germline. Moreover, previous studies demonstrated that reducing VITs in wild-type animals increases both lifespan and lipid accumulation, with overexpression having an opposite effect in long-lived mutants [49, 53]. Both RNA-Seq and qPCR experiments showed that the expression of all six *vit* genes was upregulated in *pry-1* mutants. We also observed a similar trend in *vit* expression in *hs*::*dNT-bar-1* animals (S9 Fig), which together with lipid defects (S8 Fig) support the possibility of *bar-1* being involved in *pry-1*-mediated *vit* transcription.

To examine whether PRY-1 signaling utilizes VITs to affect lipid levels, RNAi experiments were performed. The results showed that knocking down *vit* genes (*vit-1/2* and vit*-3*/*4*/*5*) suppressed low lipid phenotype of *pry-1* mutant animals, providing evidence that VITs play an important role in PRY-1-mediated lipid metabolism. We have also shown that such a role of VITs may not utilize lipoprotein receptors RME-2 (VIT transporter) or LRP-2 (VIT synthesis). Overall, these findings along with the role of VITs in regulating lipid levels [49], allow us to propose that PRY-1-mediated signaling involves VITs to regulate processes that depend on energy metabolism and lipid signaling [42, 43].

Although it remains unclear how VITs participate in *pry-1* signaling, one possibility may involve downregulation of the autophagy pathway. Manipulating VIT levels has been shown to affect the lifespan by altering autophagy [49]. Autophagy is a complex process that involves multiple enzymes to recycle cellular contents by converting them into usable metabolites. Despite the *pry-1* transcriptome not containing known autophagy-related candidates, the pathway may still be involved. This could be tested by examining the roles of specific autophagosome genes in lipoprotein synthesis and autophagy, which in turn should reveal a link with *pry-1*-mediated lipid metabolism.

### A potential role of PRY-1 in fatty acid synthesis

While lipid catabolism appears to be unaffected in *pry-1* mutants, the low lipid phenotype may be caused by reduced fatty acid synthesis. In agreement with this, the expression of three conserved stearoyl-CoA desaturases, *fat-5*, *fat-6*, and *fat-7*, which are involved in the synthesis of monounsaturated fatty acids such as OA [16–18] was reduced in *pry-1* mutants. Because these *fat* desaturases are regulated by *nhr-49* and *nhr-80*, we examined genetic interactions between *pry-1* and *nhr* genes. Our qPCR and RNAi experiments (S6 and S7 Figs) did not reveal a clear epistatic relationship, suggesting that *pry-1* is likely to act in parallel to these two transcription factors. Besides NHR-49 and NHR-80, the mammalian homolog of SREBP, SBP-1, is another transcription factor that regulates expression of *fat* genes [50, 54, 55]. Although our RNA-Seq dataset did not include *sbp-1*, we found the *sbp-1* transcript levels to be reduced in *pry-1(mu38)* animals (S6 Fig), suggesting that *pry-1* may interact with *sbp-1* to affect expression of *fat* genes. However, more experiments are needed to validate this regulatory relationship.

The GC-MS analysis of fatty acid composition revealed that *pry-1* is needed to maintain normal levels of every fatty acid species analyzed. A global reduction in fatty acids may affect processes that require utilization of lipids such as aging. However, the relationship between lipid levels and lifespan is not well understood. It is likely that rather than absolute levels, the quality of lipids may be more important for cellular processes [45]. We investigated this possibility using OA, one of the species involved in fatty acid signaling. Exogenous treatment with OA restored lipid levels in *pry-1* mutants. Thus, *pry-1* may play a role in maintaining the levels of beneficial fatty acids, possibly by affecting their synthesis. However, additional mechanisms are also likely to be involved, such as reduced conversion of acetyl-CoA to saturated fatty acid (palmitate), lower synthesis of diglycerides, and increased peroxisomal β-oxidation (S2 Fig). It would therefore be worthwhile to examine these possibilities in future studies.

It is also unclear whether PRY-1 is required in one or more tissues to maintain lipid levels. In preliminary studies using *pry-1*::*gfp* transgenic animals, we observed fluorescence in several tissues including seam cells, neuronal cells, muscles, and hypodermis. This suggests a broader requirement of PRY-1 in *C*. *elegans*, however more experiments are needed to fully understand its tissue-specific role in lipid regulation.

Our study provides the first evidence of PRY-1/Axin function in lipid metabolism. The involvement of lipids in age-related disorders in humans, as well as animal models, is well documented. Genetic and acquired lipid diseases are associated with the loss of subcutaneous fat, accumulation of visceral and ectopic fat, and metabolic syndromes such as insulin resistance, glucose intolerance, dyslipidemia, and hypertension [56]. In addition, Yang et al. showed that Axin expression in mice contributes to an age-related increase in adiposity in thymic stromal cells [10]. Although the role of PRY-1/Axin in fat storage needs to be investigated further, whether Axin family members play roles in any of these lipid-related diseases remains unknown. Therefore, the findings that PRY-1/Axin is necessary for the regulation of lipid levels provide a unique opportunity to investigate the role of Axin signaling in age-related lipid metabolism.

## Supporting information

S1 Fig Expression profile of WNT ligands, receptors, and target genes. (A-C) Developmental expression patterns of known WNT ligands, receptors and target genes from published microarray sources (see Methods). (D) qPCR validations of selected WNT target genes during the L1 and L4 stages. Each data sample represents the mean of two replicates and error bar represents the SEM. *p < 0.01.
(JPG)

S2 Fig Overview of lipid metabolism and genes with altered expression in *pry-1(mu38)*. The lipid anabolic and catabolic pathway is adapted from a previously published study [57, 58]. Lipid anabolic processes involve initiation, desaturation and elongation of fatty-acid (FA), followed by triglyceride (TAG) formation. Initiation involves conversion of Acetyl CoA to the saturated fatty-acid (SFA) Palmitate (C16:0). Elongase (*elo*) and desaturase (*fat*) enzymes act on Palmitate to modify it to long chain mono- and poly-unsaturated fatty acids (MUFAs and PUFAs, respectively). MUFAs and PUFAs are collectively termed as free fatty acids (FFAs). The FFAs are linked with glycerol 3-phosphate (Glycerol 3P) to produce lysophosphatidic acid (LPA) and phosphatidic acid (PA). PA and monoglycerides (MAG) serve as building blocks of diglycerides (DAG) synthesis. DAGs are converted into neutral lipids (TAGs). Lipid catabolism begins with the breakdown of TAGs into DAGs by ATGL-1, and other lipases and lipase-like enzymes (abbreviated as ‘*lipl’* and ‘*lips’*) to release FFAs. FFAs are further broken down to Acetyl CoA through peroxisomal- and mitochondrial- β-oxidation and release energy. Putative genes involved in lipid metabolism are shown at the appropriate step. Genes with altered expression in *pry-1(mu38)* are highlighted in blue (DOWN) and red (UP).
(JPG)

S3 Fig Quantification of lipids in *vit* and *pry-1* mutants. (A) Representative images of wild-type (WT) and *vit* mutant animals stained with Oil Red O. Refer to Fig 3 for lipid quantification in these worms. (B) Representative images of *mdt-28p*::*mCherry* and *pry-1(mu38)*; *mdt-28p*::*mCherry* animals treated with *gfp* (control) and *vit* RNAi. (C,D) Histograms showing quantification of fluorescence intensity. Data represents the mean of two replicates and error bar represents the SEM. *p < 0.05, **p < 0.01.
(JPG)

S4 Fig *vit-1* RNAi causes almost complete elimination of *vit-2* transcripts. qPCR analysis of *vit-2* in the *pry-1(mu38)* day 3 mutants after adult specific *vit-1* RNAi knockdown. Data represents the mean of three replicates and error bar represents the SEM. *p < 0.01.
(JPG)

S5 Fig Relative fatty acid abundance in *pry-1(mu38)*. (A) Relative abundance of selected fatty acid species expressed in percentage of total fatty acid as determined by GC-MS analysis. *pry-1* mutants have marginally lower levels of C15:0, C16:0 and higher levels of C20:1, C22:0 than N2 (marked with star, *p* < 0.05). Error bar represents the standard deviation. (B, C) A representative GC-MS Total Ion Chromatogram (TIC) traces of populations of the N2 and *pry-1(mu38)* worms, respectively. The peaks corresponding to fatty acid species are identified.
(JPG)

S6 Fig *sbp-1* is downregulated in *pry-1(mu38)*. qPCR analysis of *nhr-49*, *nhr-80* and *sbp-1* genes in *pry-1(mu38)* animals at the L1 stage. Data represents the mean of two replicates and error bar represents the SEM. *p < 0.05, *p < 0.01.
(JPG)

S7 Fig Genetic interaction analysis of *pry-1* with *nhr-49* and *nhr-80*. (A) Representative images after RNAi knockdown of *nhr-49* and *nhr-80* in wild-type and *pry-1(mu38)* animals. (B) Lipid quantification after *nhr-49* and *nhr-80* RNAi. (C) Representative images of *nhr-49* and *nhr-80* mutant animals fed with *gfp* (control) and *pry-1* RNAi bacteria. (D) Lipid quantification in wild-type, *nhr-49*, and *nhr-80* mutants. Data represents the mean of at least two replicates and error bar represents the SEM. n > 30, *p < 0.05.
(JPG)

S8 Fig Lipid staining and quantification in *hs*::*dNT-bar-1* animals. (A) Representative DIC images of N2 and *hs*::*dNT-bar-1* at 20 °C, after heat shock at 30 °C for 12hrs and 38 °C for 30 minutes, stained with Oil Red O. (B) Quantification of total lipids in *hs*::*dNT-bar-1* animals after heat shock treatments. Data represents the mean of at least two replicates and error bar represents the SEM. n > 50 for each trial*; p* < 0.01 for all mutants compared to the control (marked with *).
(JPG)

S9 Fig qPCR analysis of *vit* genes in *hs*::*dNT-bar-1* animals. (A) qRT-PCR of *vit* genes at L1 stage in *hs*::*dNT-bar-1* mutants after heat shock at 38 °C for 30 min. Data represents the mean of at least two replicates and error bar represents the SEM. The dotted horizontal line marks the control which is normalized to one. *p < 0.01.
(JPG)

S1 Table List of primers used in this study. (PDF)

S2 Table mRNA transcripts mapped to the *C*. *elegans* genome. (PDF)

S3 Table An Excel spreadsheet listing differentially regulated genes. (XLSX)

S4 Table An Excel spreadsheet showing GO-term enrichment. (XLSX)

S5 Table An Excel spreadsheet showing transcriptome comparison. (XLSX)

S6 Table An Excel spreadsheet showing analysis of the shared genes based on expression trends. (XLSX)

S7 Table Conservation of *vit* gene sequences used in RNAi experiments. RNAi clones are from the Ahringer library. ns: no significant identity observed.
(PDF)
