# Supplementary figures and images for: PRY-1/Axin signaling regulates lipid metabolism in Caenorhabditis elegans
Source: PLoS One. 2018 Nov 7;13(11):e0206540. doi: 10.1371/journal.pone.0206540 (PMC6221325; doi:10.1371/journal.pone.0206540)

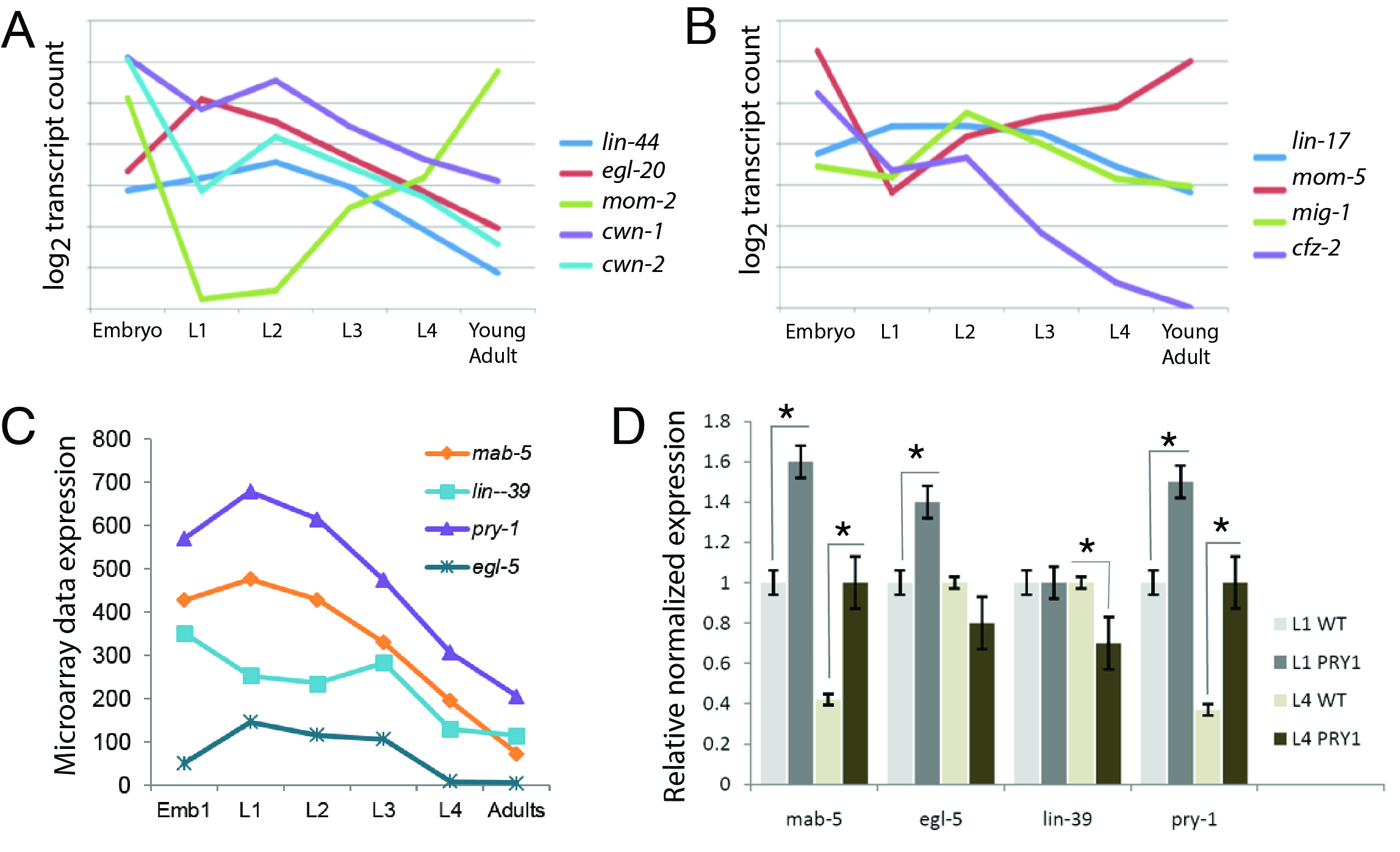

Supplement: S1 Fig — (A-C) Developmental expression patterns of known WNT ligands, receptors and target genes from published microarray sources (see Methods). (D) qPCR validations of selected WNT target genes during the L1 and L4 stages. Each data sample represents the mean of two replicates and error bar represents the SEM. *p < 0.01. (JPG) [file pone.0206540.s001.jpg]

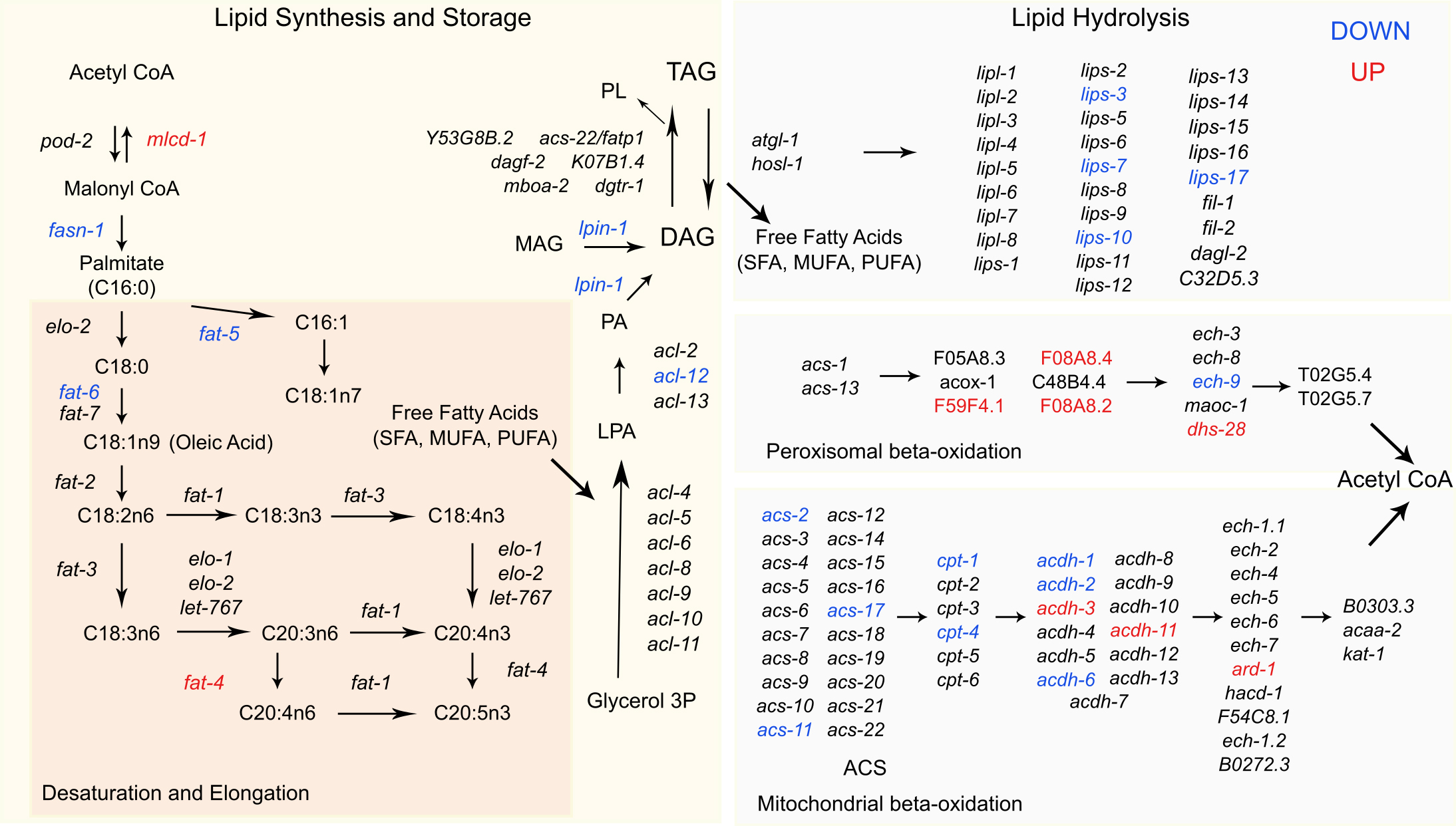

Supplement: S2 Fig — The lipid anabolic and catabolic pathway is adapted from a previously published study [57, 58]. Lipid anabolic processes involve initiation, desaturation and elongation of fatty-acid (FA), followed by triglyceride (TAG) formation. Initiation involves conversion of Acetyl CoA to the saturated fatty-acid (SFA) Palmitate (C16:0). Elongase (elo) and desaturase (fat) enzymes act on Palmitate to modify it to long chain mono- and poly-unsaturated fatty acids (MUFAs and PUFAs, respectively). MUFAs and PUFAs are collectively termed as free fatty acids (FFAs). The FFAs are linked with glycerol 3-phosphate (Glycerol 3P) to produce lysophosphatidic acid (LPA) and phosphatidic acid (PA). PA and monoglycerides (MAG) serve as building blocks of diglycerides (DAG) synthesis. DAGs are converted into neutral lipids (TAGs). Lipid catabolism begins with the breakdown of TAGs into DAGs by ATGL-1, and other lipases and lipase-like enzymes (abbreviated as ‘lipl’ and ‘lips’) to release FFAs. FFAs are further broken down to Acetyl CoA through peroxisomal- and mitochondrial- β-oxidation and release energy. Putative genes involved in lipid metabolism are shown at the appropriate step. Genes with altered expression in pry-1(mu38) are highlighted in blue (DOWN) and red (UP). (JPG) [file pone.0206540.s002.jpg]

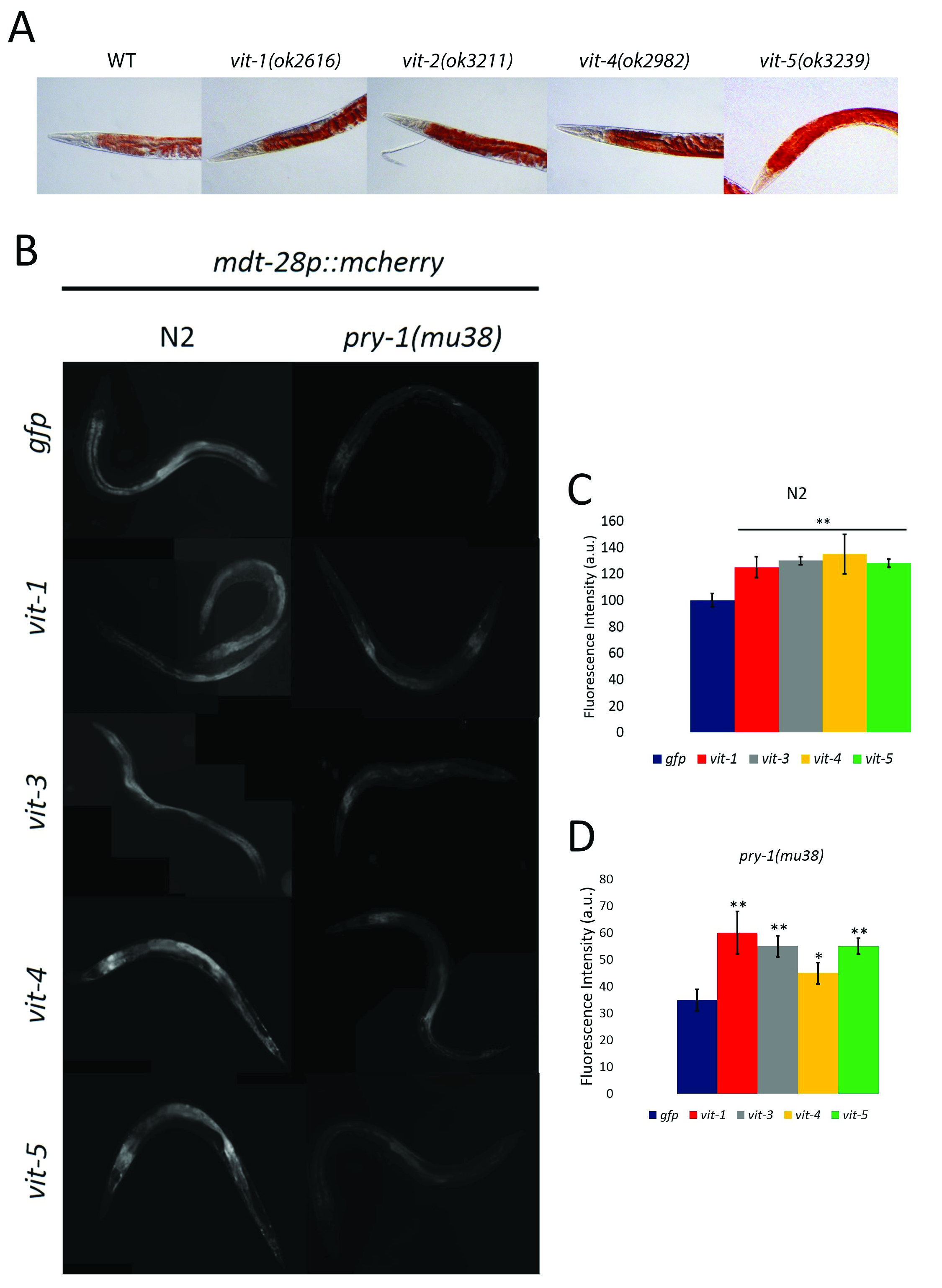

Supplement: S3 Fig — (A) Representative images of wild-type (WT) and vit mutant animals stained with Oil Red O. Refer to Fig 3 for lipid quantification in these worms. (B) Representative images of mdt-28p::mCherry and pry-1(mu38); mdt-28p::mCherry animals treated with gfp (control) and vit RNAi. (C,D) Histograms showing quantification of fluorescence intensity. Data represents the mean of two replicates and error bar represents the SEM. *p < 0.05, **p < 0.01. (JPG) [file pone.0206540.s003.jpg]

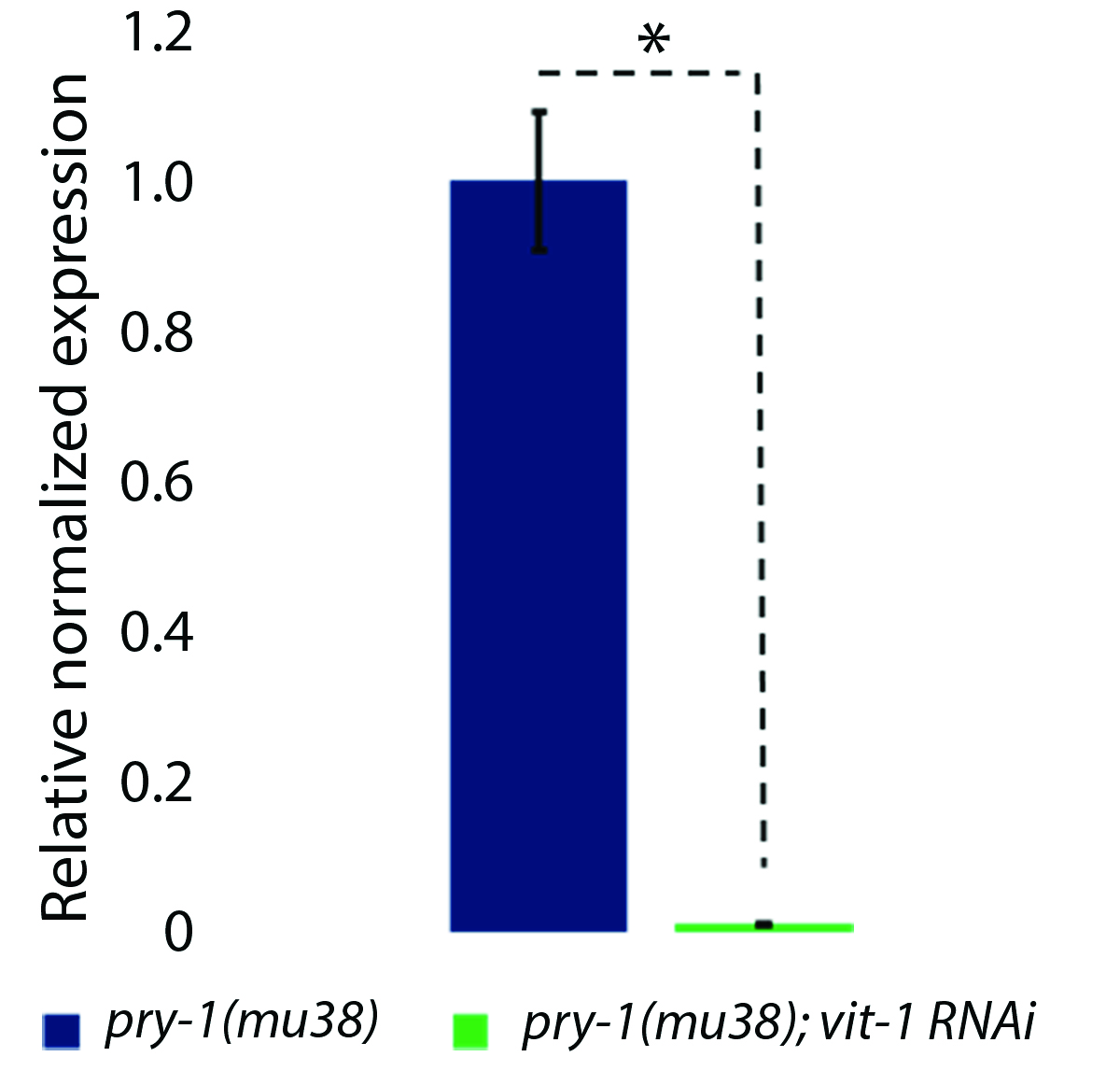

Supplement: S4 Fig — qPCR analysis of vit-2 in the pry-1(mu38) day 3 mutants after adult specific vit-1 RNAi knockdown. Data represents the mean of three replicates and error bar represents the SEM. *p < 0.01. (JPG) [file pone.0206540.s004.jpg]

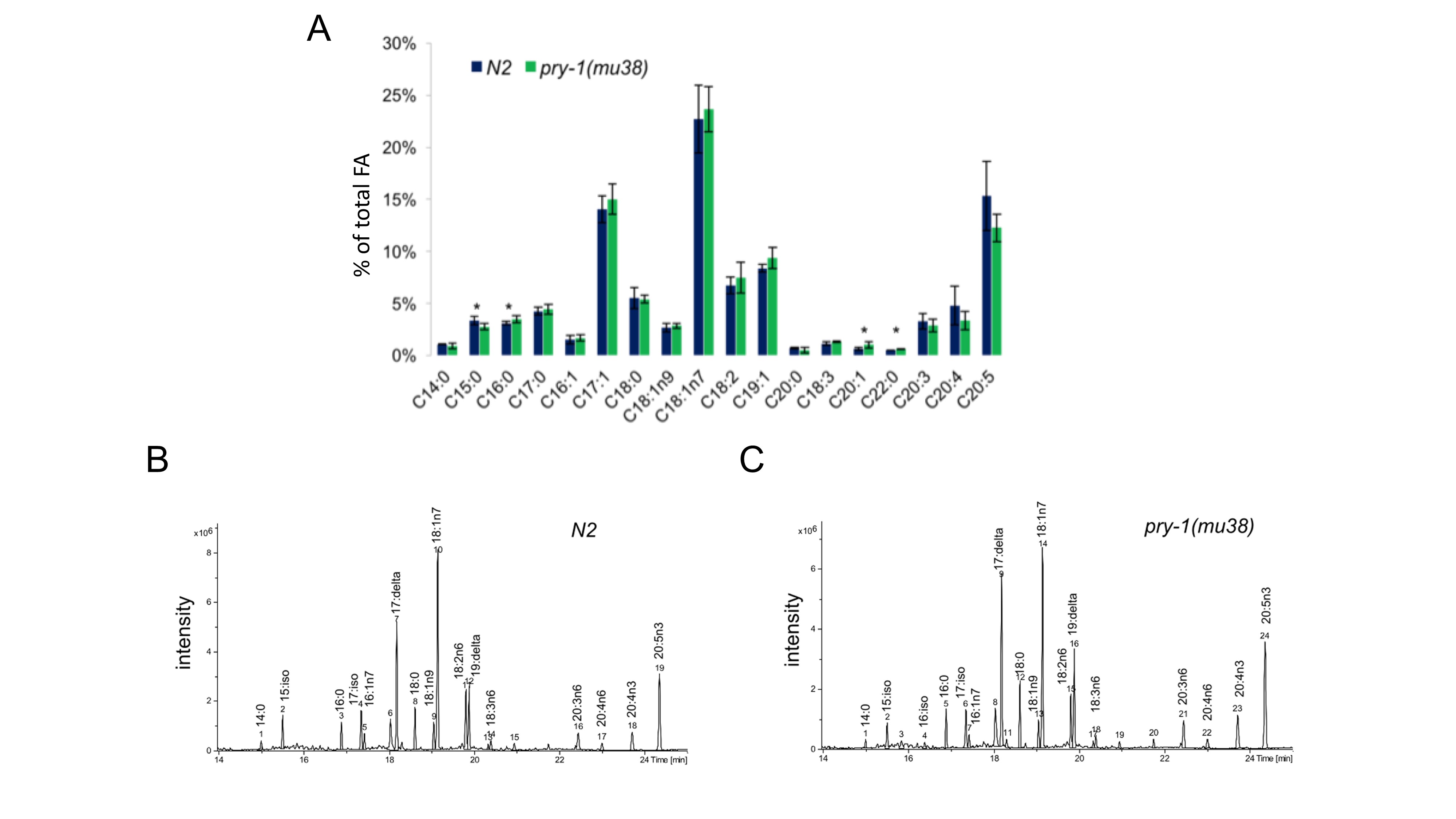

Supplement: S5 Fig — (A) Relative abundance of selected fatty acid species expressed in percentage of total fatty acid as determined by GC-MS analysis. pry-1 mutants have marginally lower levels of C15:0, C16:0 and higher levels of C20:1, C22:0 than N2 (marked with star, p < 0.05). Error bar represents the standard deviation. (B, C) A representative GC-MS Total Ion Chromatogram (TIC) traces of populations of the N2 and pry-1(mu38) worms, respectively. The peaks corresponding to fatty acid species are identified. (JPG) [file pone.0206540.s005.jpg]

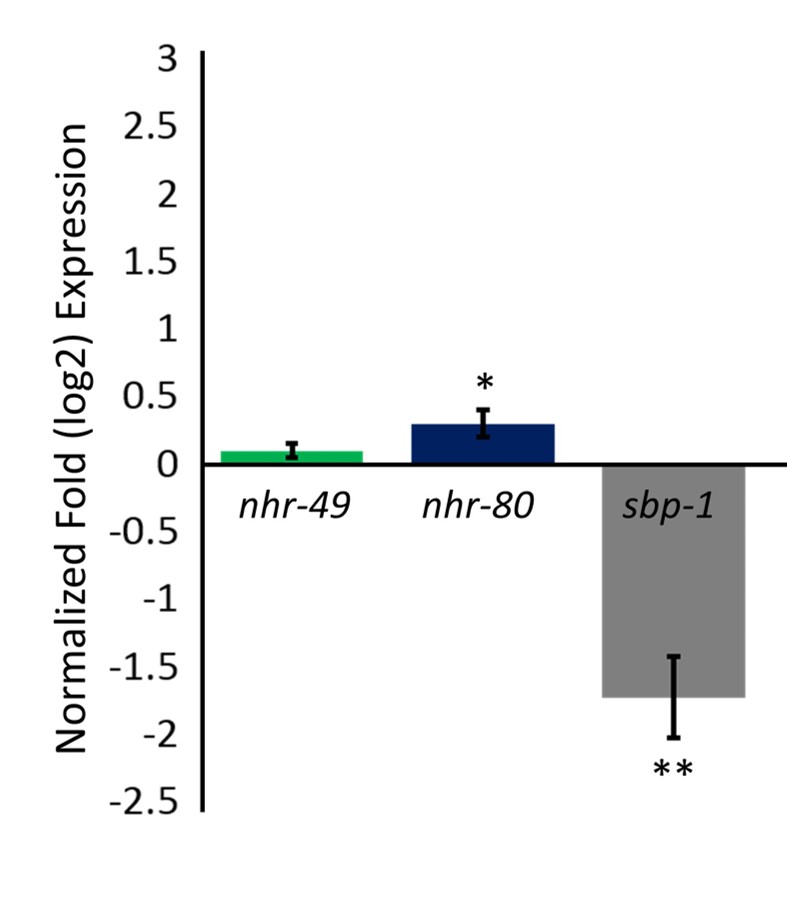

Supplement: S6 Fig — qPCR analysis of nhr-49, nhr-80 and sbp-1 genes in pry-1(mu38) animals at the L1 stage. Data represents the mean of two replicates and error bar represents the SEM. *p < 0.05, *p < 0.01. (JPG) [file pone.0206540.s006.jpg]

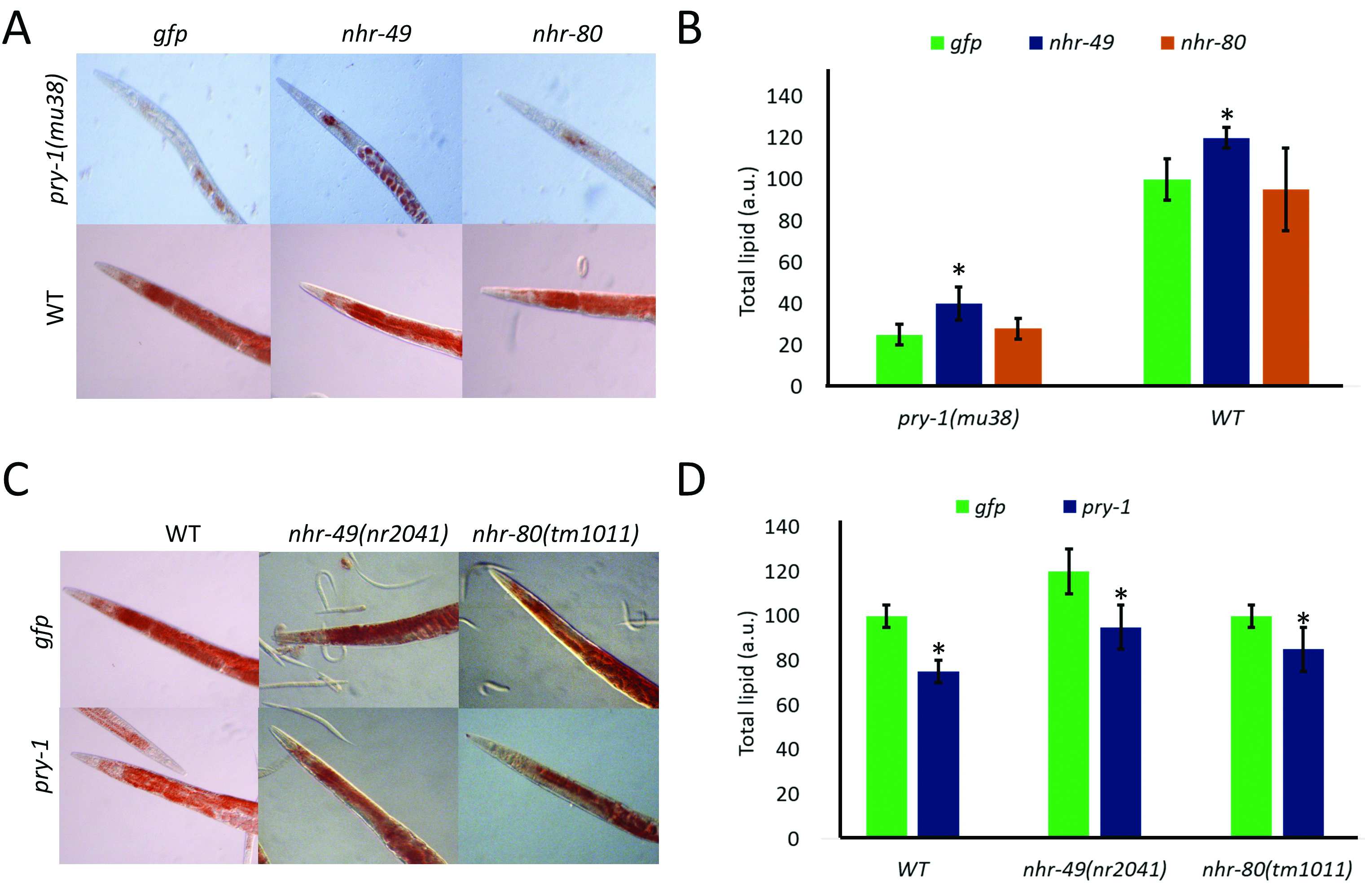

Supplement: S7 Fig — (A) Representative images after RNAi knockdown of nhr-49 and nhr-80 in wild-type and pry-1(mu38) animals. (B) Lipid quantification after nhr-49 and nhr-80 RNAi. (C) Representative images of nhr-49 and nhr-80 mutant animals fed with gfp (control) and pry-1 RNAi bacteria. (D) Lipid quantification in wild-type, nhr-49, and nhr-80 mutants. Data represents the mean of at least two replicates and error bar represents the SEM. n > 30, *p < 0.05. (JPG) [file pone.0206540.s007.jpg]

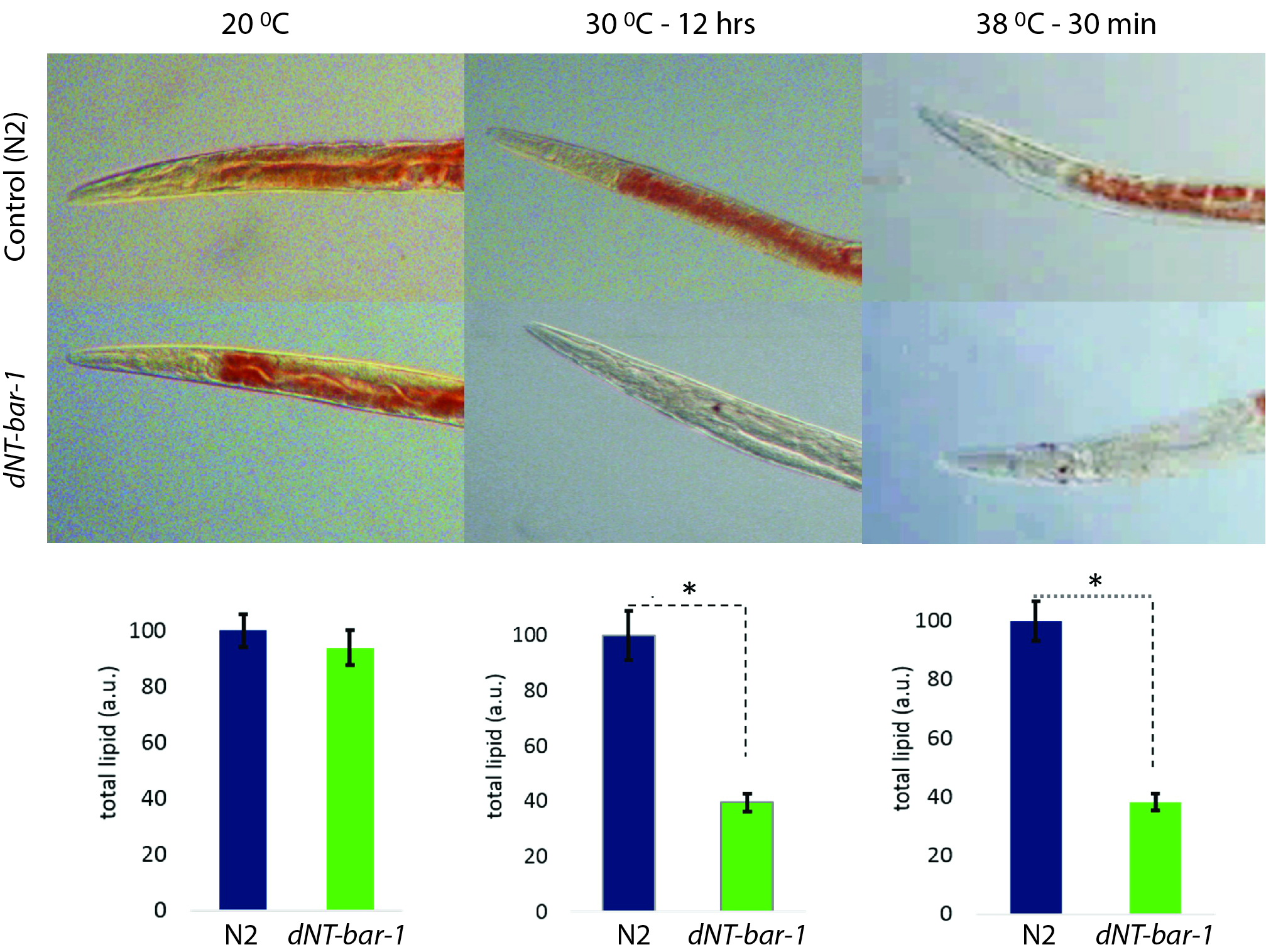

Supplement: S8 Fig — (A) Representative DIC images of N2 and hs::dNT-bar-1 at 20 °C, after heat shock at 30 °C for 12hrs and 38 °C for 30 minutes, stained with Oil Red O. (B) Quantification of total lipids in hs::dNT-bar-1 animals after heat shock treatments. Data represents the mean of at least two replicates and error bar represents the SEM. n > 50 for each trial; p < 0.01 for all mutants compared to the control (marked with *). (JPG) [file pone.0206540.s008.jpg]

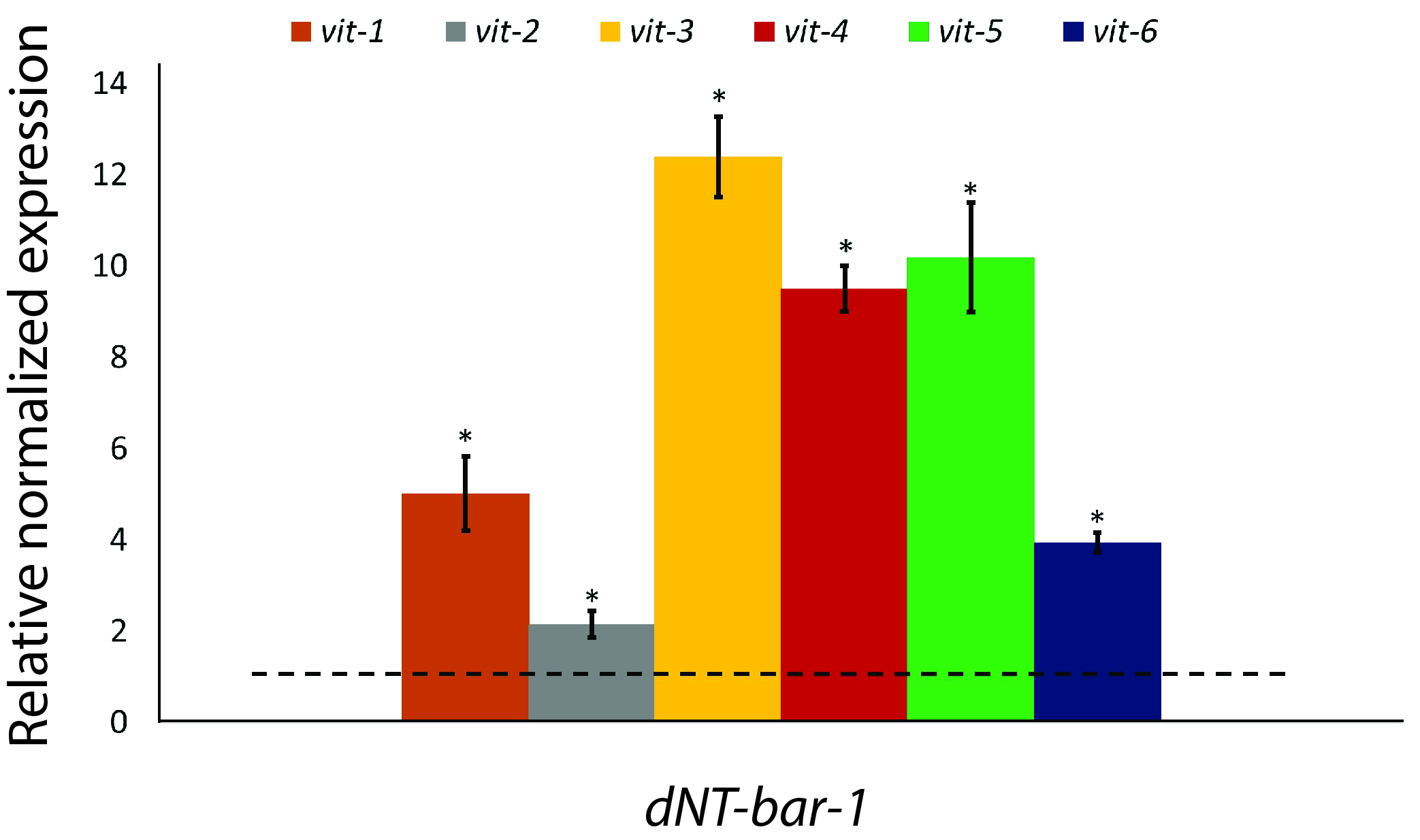

Supplement: S9 Fig — (A) qRT-PCR of vit genes at L1 stage in hs::dNT-bar-1 mutants after heat shock at 38 °C for 30 min. Data represents the mean of at least two replicates and error bar represents the SEM. The dotted horizontal line marks the control which is normalized to one. *p < 0.01. (JPG) [file pone.0206540.s009.jpg]
