# Supplementary material for: PRY-1/Axin signaling regulates lipid metabolism in Caenorhabditis elegans
Source: PLoS One. 2018 Nov 7;13(11):e0206540. doi: 10.1371/journal.pone.0206540 (PMC6221325; doi:10.1371/journal.pone.0206540)
Supplement: S1 Table — (PDF) [file pone.0206540.s010.pdf]

**S1 Table. List of primers used in this study.**

| Gene | Primer orientation (Forward, FP; Reverse, RP) | Sequence (5' to 3') |
|---|---|---|
| *pmp-3* | FP | **GL747** CTTAGAGTCAAGGGTCGCAGTGGAG |
| | RP | **GL748** ACTGTATCGGCACCAAGGAAACTGG |
| *pry-1* | FP | **GL741** CGCCAACACGAGGAGTTTGTGG |
| | RP | **GL742** TGTGATGAATGGTGGGCGGAGC |
| *pry-1* | FP | **GL1343** ATTACCCGGGCTCCGCCCACCATTCATCAC |
| | RP | **GL1344** TGCTGAGCTCGAGCCTTTCTGTGCTGCCT |
| *ptr-16* | FP | **GL853** GCAATGCTTCATCCCGATTACATCC |
| | RP | **GL854** GTGGTTTGACGATCCGTTCGGA |
| *ptr-19* | FP | **GL855** CATCAACTACCCATCAATCTGCGTG |
| | RP | **GL856** GATCCGAGACGAGAAGCAGCTTGA |
| *ptr-20* | FP | **GL857** CATTTGTGCCGACGATTTCTCAGG |
| | RP | **GL858** GTGTTGACATGAGAGACGAGGGCA |
| *grd-14* | FP | **GL876** TTTTCGTCGCCATCTCGTCT |
| | RP | **GL877** GGCATGCCTCTGGCTCATA |
| *grd-15* | FP | **GL878** GCAACGGGATGAGCAGATAGA |
| | RP | **GL879** GGGTTGCAACACATGAAGC |
| *grd-6* | FP | **GL880** TATTGCCCAGCCAAATCCAAGAGTCGT |
| | RP | **GL881** GTTGTGGTCTGTACTGTTGTTGGA |
| *grl-1* | FP | **GL882** ACTGCCACAAGATATCAGGCAT |
| | RP | **GL883** TTGTAGAGTCGGTTGCTGGG |
| *grl-16* | FP | **GL884** GCTTTGAAGAACGAGAAGGACAACC |
| | RP | **GL885** GTTTCTCTTTCCGTACCAGTTGACG |
| *grl-21* | FP | **GL886** ACGGACCAGGACCATACAGA |
| | RP | **GL887** TCACCTGATGTCATTCCCTTCT |
| *grl-4* | FP | **GL888** GGAGAGGAACATGAACGGTGA |
| | RP | **GL889** CTTGGCAGTAGGTCTCGGTG |

| | | |
|---|---|---|
| *grl-6* | FP | **GL890** GAGACCATCTTGCCCGTGTA |
| | RP | **GL891** GCAACAATCGTTCTGAGCTGG |
| *grl-13* | FP | **GL892** GGAAGAGGCAATGTCGTCCA |
| | RP | **GL893** AAATCTGGCCGTCCAACTCC |
| *vit-1* | FP | **GL872** GGTTCGCTTTGACGGATACAC |
| | RP | **GL873** AACTCGTTGGTGGACTCATC |
| *vit-2* | FP | **GL870** GACACCGAGCTCATCCGCCCA |
| | RP | **GL871** TTCCTTCTCTCCATTGACCT |
| *vit-3* | FP | **GL868** GGCTCGTGAGCAAACTGTTG |
| | RP | **GL869** TTAATAGGCAACGCAGGCGG |
| *vit-4* | FP | **GL866** TGTCAACGGACAAGAGGTTG |
| | RP | **GL867** TCCTTTGGTCCAGAGACCTTC |
| *vit-5* | FP | **GL864** GGCAATTTGTTAAGCCACAA |
| | RP | **GL865** CCTCCTTTGGTCCAGAAACCT |
| *vit-6* | FP | **GL862** AGTCGCTATTGTCGAGCGTC |
| | RP | **GL863** AGACGGAGGTCACCTTTTGC |
| *fat-4* | FP | **GL1201** ATGGATGCCACTACCGTATTCC |
| | RP | **GL1202** TCTGGTTCTTGTGTAGGGCAC |
| *fat-5* | FP | **GL1203** ATGGGTATTCCTCCTGCACAC |
| | RP | **GL1204** TCCATGAGAGGGTGGCTTTG |
| *fat-6* | FP | **GL1205** GCGCTGCTCACTATTTCGG |
| | RP | **GL1206** GGAAGTTGTGACCTCCCTCTC |
| *fat-7* | FP | **GL1207** GCGCTGCTCACTATTTTGGT |
| | RP | **GL1208** TGTGACCTCCTTCACCAACG |
| *nhr-49* | FP | **GL1209** ATCACCGACGAGATCATGCC |
| | RP | **GL1210** TCGAAACCCCCTTGAAAGCA |
| *nhr-80* | FP | **GL1211** GGTCGAATGGAAATGACACAGA |
| | RP | **GL1212** CATTCAGATCTACCTCGGTTGTG |
| *sbp-1* | FP | **GL1398** GGACCATCACAACAACCGGA |
| | RP | **GL1399** GCAGGGAGTGTAAGGTGCTT |
| *atgl-1* | FP | **GL1240** CCGACTACAAGTAAACGTGATGC |

| | | |
|---|---|---|
| | RP | **GL1241** AGATTCGGCTGGTTGAGGTG |
| *fasn-1* | FP | **GL1236** ACTGAAGGAGTTGCAGCCAT |
| | RP | **GL1237** CTCCTTGTGCCCATCAGTGT |
| *mlcd-1* | FP | **GL1234** TCACAAAAAGAGGAGCACCG |
| | RP | **GL1235** ATACCAGAAAGACCAGGCTGTG |
| *mab-5* | FP | **GL737** AGCATGTATCCTGGATGGACAGGCG |
| | RP | **GL738** TGCTGAAGCAGATGTGCCGGATG |
| *egl-5* | FP | **GL745** ACGGCTGGCCACAGAACTACAAC |
| | RP | **GL746** AGTTGGGCCACGCCGTATTC |
| *lin-39* | FP | **GL739** ACTGCACCGCCTGAATTCTTATCC |
| | RP | **GL740** TGGAAGCACCTGGAAGGAGACG |
