# Supplementary material for: PRY-1/Axin signaling regulates lipid metabolism in Caenorhabditis elegans
Source: PLoS One. 2018 Nov 7;13(11):e0206540. doi: 10.1371/journal.pone.0206540 (PMC6221325; doi:10.1371/journal.pone.0206540)
Supplement: S2 Table — (PDF) [file pone.0206540.s011.pdf]

S2 Table. mRNA transcripts mapped to the *C. elegans* genome.

	N2_1	*N2_2*	*pry-1_1*	*pry-1_2*
Number of input reads	31398474	38382323	36861495	35886365
Average input read length	194	193	193	193
UNIQUE READS				
Number of reads	29693460	35944075	35124604	34111824
Uniquely mapped reads %	94.57%	93.65%	95.29%	95.06%
Average mapped length	192.8	192.79	192.66	192.6
Mismatch rate per base, %	0.11%	0.11%	0.11%	0.11%
READS MAPPED TO MULTIPLE LOCI				
Number of reads	1182683	1790500	1131080	1182083
% of reads	3.77%	4.66%	3.07%	3.29%
Number of reads mapped to several loci	75341	122959	79013	85400
% of reads mapped to several loci	0.24%	0.32%	0.21%	0.24%
UNMAPPED READS				
% of reads unmapped: many mismatches	0.00%	0.00%	0.00%	0.00%
% of reads unmapped: too short	1.39%	1.33%	1.40%	1.38%
% of reads unmapped: other	0.03%	0.03%	0.03%	0.03%
