# Supplementary material for: PRY-1/Axin signaling regulates lipid metabolism in Caenorhabditis elegans
Source: PLoS One. 2018 Nov 7;13(11):e0206540. doi: 10.1371/journal.pone.0206540 (PMC6221325; doi:10.1371/journal.pone.0206540)
Supplement: S7 Table — RNAi clones are from the Ahringer library. ns: no significant identity observed. (PDF) [file pone.0206540.s016.pdf]

**S7 Table. Conservation of *vit* gene sequences used in RNAi experiments.** RNAi clones are from the Ahringer library. ns: no significant identity observed.

| gene targets | *vit* gene fragments used in RNAi | | | |
|---|---|---|---|---|
| | *vit-1* | *vit-3* | *vit-4* | *vit-5* |
| *vit-1* | 100% | ns | 80% (98bp) | 80% (123bp) |
| *vit-2* | 94% (1025 bp) | ns | 80% (99bp) | 81% (123bp) |
| *vit-3* | ns | 100% | 99% (2421bp) | 94% (1125bp) |
| *vit-4* | ns | 98% (2364bp) | 100% | 97% (1075bp) |
| *vit-5* | ns | 97% (2267bp) | 98% (2397bp) | 100% |
| *vit-6* | ns | ns | ns | ns |
